# BRD4 inhibition and FXR activation, individually beneficial in cholestasis, are antagonistic in combination

**DOI:** 10.1172/jci.insight.141640

**Published:** 2020-12-08

**Authors:** Hyunkyung Jung, Jinjing Chen, Xiangming Hu, Hao Sun, Shwu-Yuan Wu, Cheng-Ming Chiang, Byron Kemper, Lin-Feng Chen, Jongsook Kim Kemper

**Affiliations:** 1 Department of Molecular and Integrative Physiology and; 2 Department of Biochemistry, School of Molecular and Cellular Biology, University of Illinois at Urbana-Champaign, Urbana, Illinois, USA.; 3 Harold C. Simmons Comprehensive Cancer Center, Department of Biochemistry, and Department of Pharmacology, University of Texas Southwestern Medical Center, Dallas, Texas, USA.; 4 Carl R. Woese Institute of Genomic Biology, University of Illinois at Urbana-Champaign, Urbana, Illinois, USA.

**Keywords:** Hepatology, Inflammation, Homeostasis, Molecular biology

## Abstract

Activation of farnesoid X receptor (FXR) by obeticholic acid (OCA) reduces hepatic inflammation and fibrosis in patients with primary biliary cholangitis (PBC), a life-threatening cholestatic liver failure. Inhibition of bromodomain-containing protein 4 (BRD4) also has antiinflammatory, antifibrotic effects in mice. We determined the role of BRD4 in FXR function in bile acid (BA) regulation and examined whether the known beneficial effects of OCA are enhanced by inhibiting BRD4 in cholestatic mice. Liver-specific downregulation of BRD4 disrupted BA homeostasis in mice, and FXR-mediated regulation of BA-related genes, including small heterodimer partner and cholesterol 7 alpha-hydroxylase, was BRD4 dependent. In cholestatic mice, JQ1 or OCA treatment ameliorated hepatotoxicity, inflammation, and fibrosis, but surprisingly, was antagonistic in combination. Mechanistically, OCA increased binding of FXR, and the corepressor silencing mediator of retinoid and thyroid hormone receptor (SMRT) decreased NF-κB binding at inflammatory genes and repressed the genes in a BRD4-dependent manner. In patients with PBC, hepatic expression of *FXR* and *BRD4* was significantly reduced. In conclusion, BRD4 is a potentially novel cofactor of FXR for maintaining BA homeostasis and hepatoprotection. Although BRD4 promotes hepatic inflammation and fibrosis in cholestasis, paradoxically, BRD4 is required for the antiinflammatory, antifibrotic actions of OCA-activated FXR. Cotreatment with OCA and JQ1, individually beneficial, may be antagonistic in treatment of liver disease patients with inflammation and fibrosis complications.

## Abstract 2

Treatment with obeticholic acid, a farnesoid X receptor agonist, or JQ1, a bromodomain-containing protein 4 inhibitor, has antiinflammatory, antifibrotic effects in cholestatic mice, but beneficial effects are lost in combination.

## Introduction

Bile acids (BAs) aid in digestion of lipid nutrients but also function as signaling molecules that profoundly influence metabolism and energy balance (1, 2). Because of detergent-like properties, elevated BA levels are toxic and lead to cholestatic liver injury with inflammation and fibrosis, which can progress further to liver failure and life-threatening liver diseases, such as primary biliary cholangitis/cirrhosis (PBC/cirrhosis) and hepatocellular carcinoma (3, 4).

Farnesoid X receptor (FXR/NR1H4) plays a central role in maintaining normal BA levels and protecting against cholestatic liver injury by transcriptional regulation of genes involved in BA synthesis, transport, and metabolism (5–7). The transcriptional function of FXR is coactivated by epigenetic proteins, including P300 acetyltransferase and brahma-related gene 1 chromatin remodeler (8–11). In addition to its gene activation function, agonist-activated FXR also has direct gene repression function revealed by published ChIP-Seq studies (12). Indeed, FXR antagonizes inflammatory actions of NF-κB (6, 13–15) and mediates repression of autophagy network genes to maintain cellular homeostasis (16, 17). As such, there is great interest in FXR as a therapeutic target for treating chronic liver diseases, including cholestasis, cirrhosis, and nonalcoholic steatohepatitis (NASH) (3, 4, 18). An FXR agonist, obeticholic acid (OCA), was recently approved by the FDA for treatment of patients with PBC who did not respond adequately to ursodeoxycholic acid (UDCA), the only drug approved previously (3, 4, 19). OCA also has antiinflammatory effects in rodent models of NASH and patients with NASH (20). In patients with NASH in recent phase 3 studies, OCA reduced liver fibrosis but was not approved on an accelerated basis by the FDA, and additional safety and clinical efficacy data were requested. Despite its exciting therapeutic potential, the molecular mechanisms of the antiinflammatory, antifibrotic actions of OCA remain unclear.

Epigenetic regulation by coordinated actions of epigenetic writer, reader, and eraser proteins plays a crucial role in maintaining homeostasis in response to environmental cues. Bromodomain-containing protein 4 (BRD4) is an epigenetic reader protein and, with BRD2, BRD3, and BRDT, belongs to the bromodomain extra-terminal (BET) family (21). BRD4 binds to acetylated histones and nonhistone proteins and activates gene transcription by stabilizing binding of coactivators, mediators, and RNA polymerase II (21–25). Intriguingly, BRD4 also has repressor functions in regulation of human papilloma virus genes and autophagy (21, 26). BRD4 plays an important role in many biological processes, including coactivating NF-κB proinflammatory functions (25) and inducing cell cycle and tumorigenic genes (27). Not surprisingly, BRD4 has emerged as a potential drug target for numerous diseases, including cancer and metabolic diseases, such as cholestasis and NASH (27–30). Intriguingly, inhibition of BRD4 by a small molecule inhibitor, such as JQ1 or I-BET151, improves NASH fibrosis and cardiac fibrosis in rodent models (29–32). The antiinflammatory, antifibrotic actions of both OCA and JQ1 thus suggest that cotreatment with OCA and a BRD4 inhibitor might be additive or synergistic in treating liver disease with inflammation and fibrosis complications.

Previously, we reported that acetylation of histone H3 at K9/14 at the small heterodimer partner (*Shp*) promoter by P300 acetyltransferase is required for FXR-mediated induction of *Shp*, a key regulator of BA metabolism (8, 10, 11). Since BRD4 is known to interact with P300 and binds to acetylated histones (21, 25, 33), we have examined whether BRD4 has a role in the FXR induction of *Shp* in the regulation of BA levels in mice. We further tested whether the known antiinflammatory, antifibrotic effects of OCA are enhanced by BRD4 in cholestatic mice. We show that BRD4 is a critical cofactor of FXR in the maintenance of BA homeostasis and hepatoprotection. Surprisingly, in cholestatic mice, inhibition of BRD4 improves hepatic inflammation and fibrosis (30–32), but paradoxically, OCA-induced antiinflammatory, antifibrotic effects are lost by cotreatment with the BRD4 inhibitor, JQ1, or liver-specific downregulation of BRD4.

## Results

### Liver-specific downregulation of BRD4 disrupts BA homeostasis in mice.

**Figure 1:**
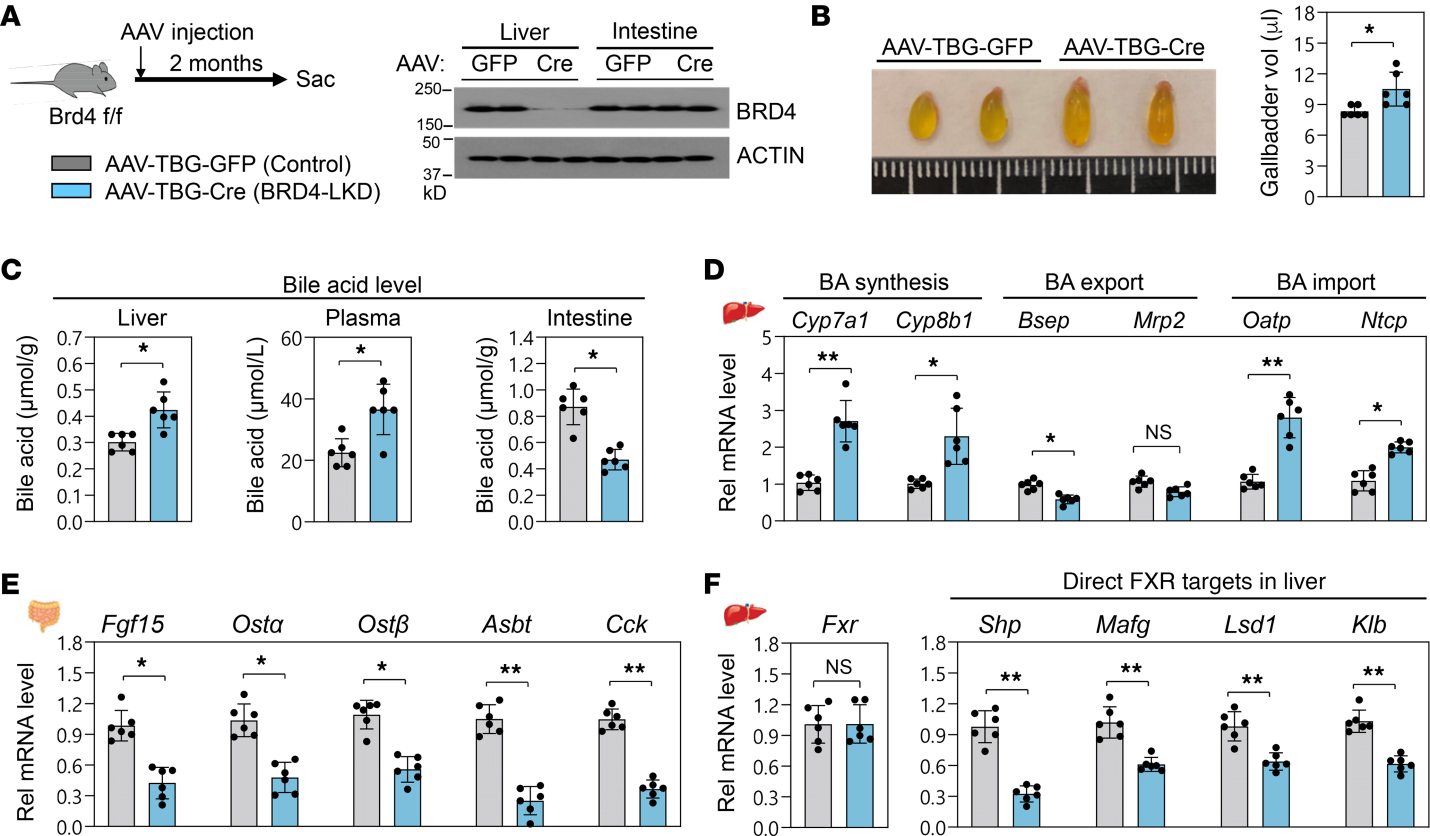
Liver-specific downregulation of BRD4 disrupts BA homeostasis in mice. For liver knockdown (LKD) of BRD4, *Brd4*-floxed mice were infected with hepatocyte-specific AAV-TBG-Cre, or AAV-TBG-GFP as a control, and after 8 weeks were fasted for 4 hours before sacrifice. (**A**) Experimental outline (left) and hepatic and intestinal BRD4 protein levels measured by immunoblot (right). (**B**) Representative images of gallbladders (left) and gallbladder volumes (right). (**C**) Hepatic, plasma, and intestinal BA levels. (**D**–**F**) Levels of the indicated mRNAs in the intestine (**E**) or liver (**D** and **F**) measured by reverse transcription quantitative PCR (RT-qPCR). (**B**–**F**) Mean and SD are plotted. Statistical significance was determined by an unpaired 1-tailed Student’s *t* test (*r* *=* 6 mice), *P *<* 0.05; **P *<* 0.01; NS, not significant. AAV, adeno-associated virus.

To examine whether hepatic BRD4 has a novel function in BA regulation, BRD4 was downregulated specifically in the liver (BRD4 liver knockdown; BRD4-LKD mice) by infection of BRD4-floxed mice with hepatocyte-targeting AAV-TBG-Cre (refs. 5, 34; and Figure 1A). The gallbladder size and volume were markedly increased in BRD4-LKD mice compared with control mice (Figure 1B), and BA levels in liver and plasma were increased, whereas intestinal BA levels were decreased (Figure 1C). Consistent with increased liver BA levels in BRD4-LKD mice, hepatic mRNA levels of the BA synthetic genes, *Cyp7a1* and *Cyp8b1*, and the BA import transporters, *Ntcp* and *Oatp*, were increased, whereas those of BA export transporters, *Bsep* and *Mrp2*, were decreased and unchanged, respectively (Figure 1D). Liver-specific downregulation of BRD4 also decreased mRNA levels of *Fgf15*, an FXR-induced gut hormone that represses hepatic BA synthesis (35), and of intestinal genes involved in enterohepatic BA recycling (36), *Osta*, *Ostb*, and *Asbt*, and in gallbladder emptying, *Cck1* (Figure 1E). These results suggest that liver-specific downregulation of BRD4 disrupts BA homeostasis in mice.

FXR regulates BA levels by transcriptional control of numerous BA-related genes (37). Although hepatic mRNA levels of *Fxr* were not changed in BRD4-LKD mice, hepatic mRNA levels of direct FXR target genes important for repression of BA synthesis, such as *Shp*, *Mafg*, *Lsd1* (37, 38), *Klb* (39), which encodes β-Klotho, the essential coreceptor for FGF15 (Figure 1F); and *Bsep* (Figure 1D), were all downregulated. These findings reveal a potential role for BRD4 in regulating BA levels, possibly through transcriptional coactivation of FXR.

### GW4064 treatment induces the interaction of FXR with BRD4.

**Figure 2:**
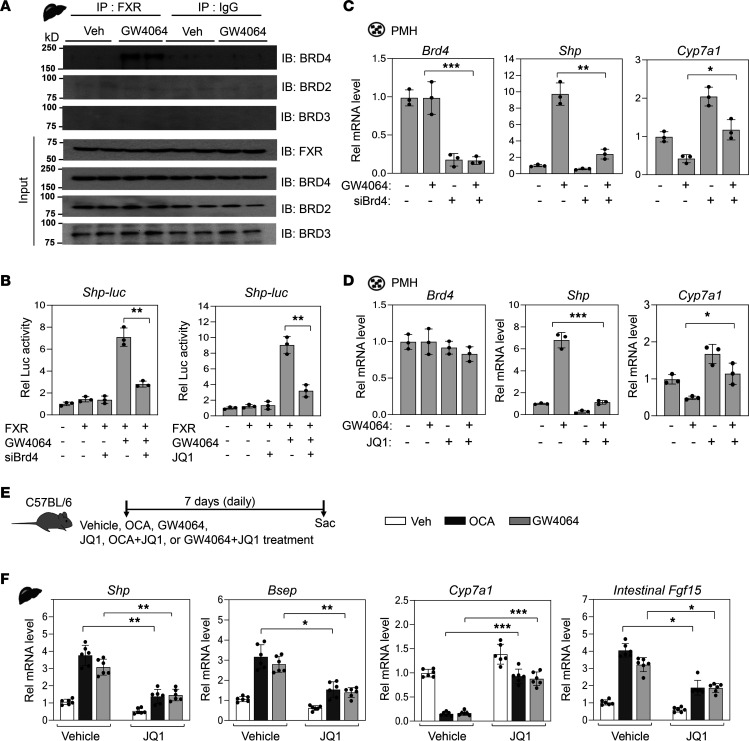
BRD4 coactivates FXR for induction of *Shp*. (**A**) Co-IP: C57BL/6 mice were treated with GW4064 or vehicle for 1 hour. The indicated proteins in anti-FXR immune complexes from liver whole-cell extracts or input levels detected by immunoblot. (**B**) Reporter assay: HepG2 cells were infected with Ad-FXR, transfected with reporter plasmids for *Shp-luc* and β-galactosidase, and treated with GW4064, JQ1, or *Brd4* siRNA (siBrd4) as indicated. Luciferase activities normalized to β-galactosidase levels. (**C** and **D**) RT-qPCR: PMHs were treated with siBrd4, JQ1, or GW4064 as indicated, and levels of mRNA for *Brd4*, *Shp*, and *Cyp7a1* in hepatocytes were determined by RT-qPCR. (**E**–**F**) C57BL/6 mice were treated daily with 30 mg/kg GW4064, 10 mg/kg OCA, or 50 mg/kg JQ1 for 7 days as indicated. (**E**) Experimental outline. (**F**) Levels of indicated mRNAs. (**B**–**D** and **F**) Mean ± SD. Statistical significance was determined by 1-way (**B**, *r* *=* 3 culture dishes) or 2-way ANOVA (**C** and **D**, *r* *=* 3 culture dishes; **F**, *r* *=* 6–8 mice), *P *<* 0.05, **P *<* 0.01, ***P *<* 0.001.

To test whether BRD4 coactivates FXR function, mice were briefly treated with an FXR agonist, GW4064, and the interaction of FXR with the BET proteins, BRD2, BRD3, and BRD4, in liver extracts was examined by co-IP assays. Treatment with GW4064 dramatically increased the interaction of FXR with BRD4 but not with BRD2 or BRD3 (Figure 2A). In glutathione-*S*-transferase pull-down assays, direct interaction between FXR and BRD4 was undetectable, while direct interaction of FXR with P300, a known FXR coactivator (8), was observed (Supplemental Figure 1; supplemental material available online with this article; https://doi.org/10.1172/jci.insight.141640DS1). These results suggest that activation of FXR induces an indirect interaction with BRD4 in a protein complex.

### BRD4 is required for FXR-mediated transactivation of Shp.

We examined whether BRD4 acts as a transcriptional coactivator of FXR using *Shp* as a model gene. BRD4 function was blocked by either siRNA-mediated downregulation or treatment with JQ1, a small molecule inhibitor of BET proteins, particularly BRD4 (27). JQ1 acts as an acetyl-Lys mimetic and inhibits binding of the BET proteins to acetyl-Lys residues (27). Treatment with GW4064 increased luciferase activity of the *Shp* promoter-reporter, and either siRNA-mediated downregulation of *Brd4* or inhibition of BRD4 by JQ1 largely abolished the increase (Figure 2B). Tandem bromodomain1 (BD1) and BD2 are critical for BRD4 interaction with transcription factors (23, 25). Overexpression of BRD4 increased FXR-mediated luciferase activity, but that of a BRD4 mutant lacking BD1 or BD2 domains did not (Supplemental Figure 2).

We next examined whether BRD4 regulates expression of endogenous *Shp* and cholesterol 7 alpha-hydroxylase (*Cyp7a1*), a direct SHP target and key BA synthetic gene, in primary mouse hepatocytes (PMHs). As expected, treatment with GW4064 resulted in substantial increases in *Shp* mRNA levels and decreases in *Cyp7a1* mRNA levels, and these GW4064-mediated effects were lost by downregulation or inhibition of BRD4 (Figure 2, C and D). These results suggest that BRD4 acts as a novel coactivator of FXR in transcriptional induction of *Shp*.

### OCA-activated FXR interacts with BRD4 to regulate BA-related genes.

OCA, also known as INT-747, is a semisynthetic FXR agonist that has been approved for treatment of patients with PBC (3, 4, 19). Consistent with the GW4064 results above (Figure 2A), OCA treatment of mice also increased the interaction of FXR with BRD4 in liver extracts (Supplemental Figure 3). In PMHs, OCA increased *Shp* mRNA levels and decreased *Cyp7a1* mRNA levels, effects that were blunted by JQ1 (Supplemental Figure 4). In mice, treatment with OCA or GW4064 increased mRNA levels of direct FXR target genes, *Shp*, *Bsep*, and *Fgf15*, and decreased *Cyp7a1* mRNA levels, and these OCA-mediated effects were substantially reduced by JQ1 (Figure 2, E and F). These results demonstrate that activation of FXR signaling by either OCA or GW4064 induces the interaction of FXR with BRD4 and increases expression of *Shp*, resulting in repression of *Cyp7a1*, in a BRD4-dependent manner.

### BRD4 is recruited to FXR-bound chromatin at the Shp promoter.

BRD4 binds to acetylated histones and nonhistone proteins to activate transcription (21, 25). Since acetylation of histone H3 at K9/14 by P300, also known as E1A-associated protein P300 (EP300), is critical for induction of *Shp* by agonist-activated FXR (8, 10, 11) and BRD4 binds to acetylated histones (21, 24), we examined whether BRD4, with FXR and P300, is recruited to the promoter of *Shp* after GW4064 treatment.

**Figure 3:**
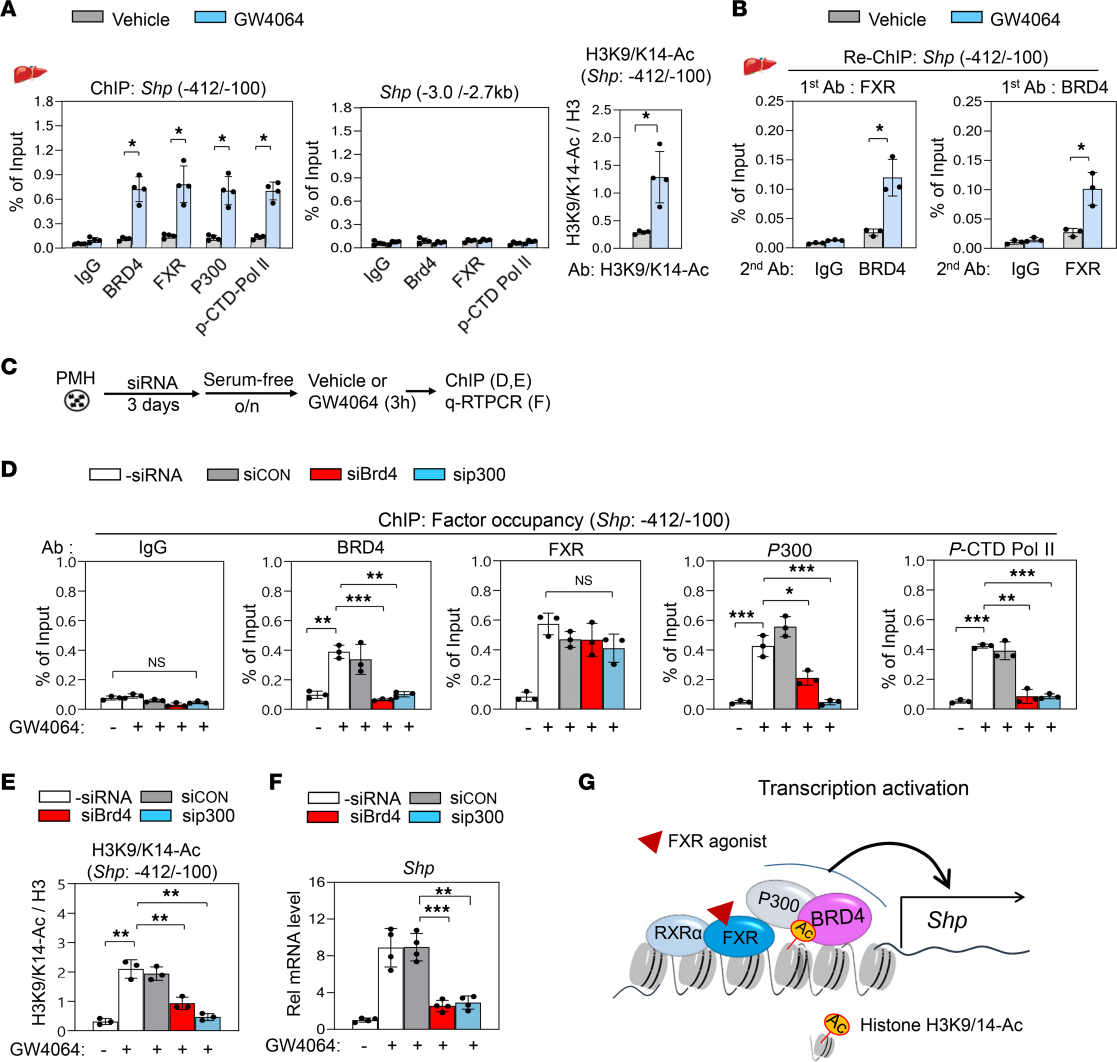
BRD4 is recruited to the FXR-bound *Shp* promoter upon GW4064 treatment. (**A** and **B**) C57BL/6 mice were treated with 30 mg/kg GW4064 or vehicle 1 hour before sacrifice. (**A**) Occupancy of the indicated proteins and histone H3K9/14-Ac levels at the *Shp* promoter by ChIP. (**B**) *Shp* promoter sequence in chromatin precipitated by FXR antibody reprecipitated with BRD4 antibody or IgG (left) or vice versa (right). (**C**–**F**) Effects of downregulation of BRD4 or P300 on factor occupancy and H3K9/14-Ac levels at the *Shp* promoter in PMHs. (**C**) Experimental outline of downregulation BRD4 and P300 (above) and mRNA levels of *Brd4* (left) and *Ep300* (right) (below). (**D**–**F**) Occupancy of the indicated factors and H3K9/14-Ac levels at the *Shp* promoter and *Shp* mRNA levels. (**G**) Model: BRD4 is critical for FXR-mediated transcription activation of Shp. Agonist-activated FXR, with RXRα, binds to *Shp* and recruits P300, resulting in increased acetylation of histone H3K9/14. BRD4 is recruited and stabilizes binding of P300 to sustain epigenetic induction of *Shp*. (**A**–**F**) Mean ± SD. Statistical significance was determined by 2-way (**A** and **B**, *r* *=* 3–4 mice) or 1-way ANOVA (**D**–**F**, *r* *=* 3–4 culture dishes), *P *<* 0.05, **P *<* 0.01, ***P *<* 0.001. o/n, overnight.

In liver ChIP assays, occupancy of BRD4, FXR, P300, and RNA polymerase II phosphorylated at the C-terminal domain (p-CTD Pol II), an indicator of active transcription (21, 23), was increased at the *Shp* promoter after GW4064 treatment (Figure 3A, left). In contrast, binding of these proteins at control regions of *Shp*, which do not contain an FXR binding motif, was not detected (Figure 3A, middle). Consistent with increased binding of P300, levels of acetylated histone H3K9/14-Ac were increased at the *Shp* promoter after GW4064 treatment (Figure 3A, right). In re-ChIP assays, GW4064 increased binding of BRD4 at FXR-bound chromatin and, conversely, binding of FXR at BRD4-bound chromatin (Figure 3B), suggesting that agonist-activated FXR and BRD4 co-occupy the *Shp* promoter. These results suggest that activation of FXR signaling by GW4064 treatment induces a functional interaction between BRD4 and FXR at the *Shp* promoter.

### BRD4 epigenetically coactivates FXR in induction of Shp.

To understand the molecular mechanism by which BRD4 coactivates FXR transcription function, BRD4 or P300 was downregulated in PMHs and ChIP assays were performed (Figure 3C and Supplemental Figure 5). GW4064 treatment increased binding of FXR, BRD4, P300, and p-CTD-Pol II (Figure 3D) and acetylated H3K9/14-Ac levels at the *Shp* promoter as expected (Figure 3E). In contrast, downregulation of BRD4 decreased binding of P300 and p-CTD-Pol II and decreased levels of H3K9/14-Ac, but FXR binding was not significantly decreased (Figure 3, D and E). Downregulation of P300 also decreased H3K9/14-Ac levels and decreased binding of BRD4 and p-CTD-Pol II, although binding of FXR was not significantly changed. Consistent with these findings, increased mRNA levels of *Shp* after GW4064 treatment were diminished by downregulation of either BRD4 or P300 (Figure 3F). These results suggest that BRD4 is not required for initial recruitment of FXR to the *Shp* promoter, but rather required for increased binding of P300 and p-CTD-Pol II and increased acetylation of histone H3 at K9/14, a gene-activating histone modification (model, Figure 3G). These data, together, demonstrate that BRD4 is a bona fide novel coactivator of FXR for epigenetic induction of *Shp*.

### OCA-induced antiinflammatory antifibrotic protective effects against cholestatic injury are BRD4 dependent.

**Figure 4:**
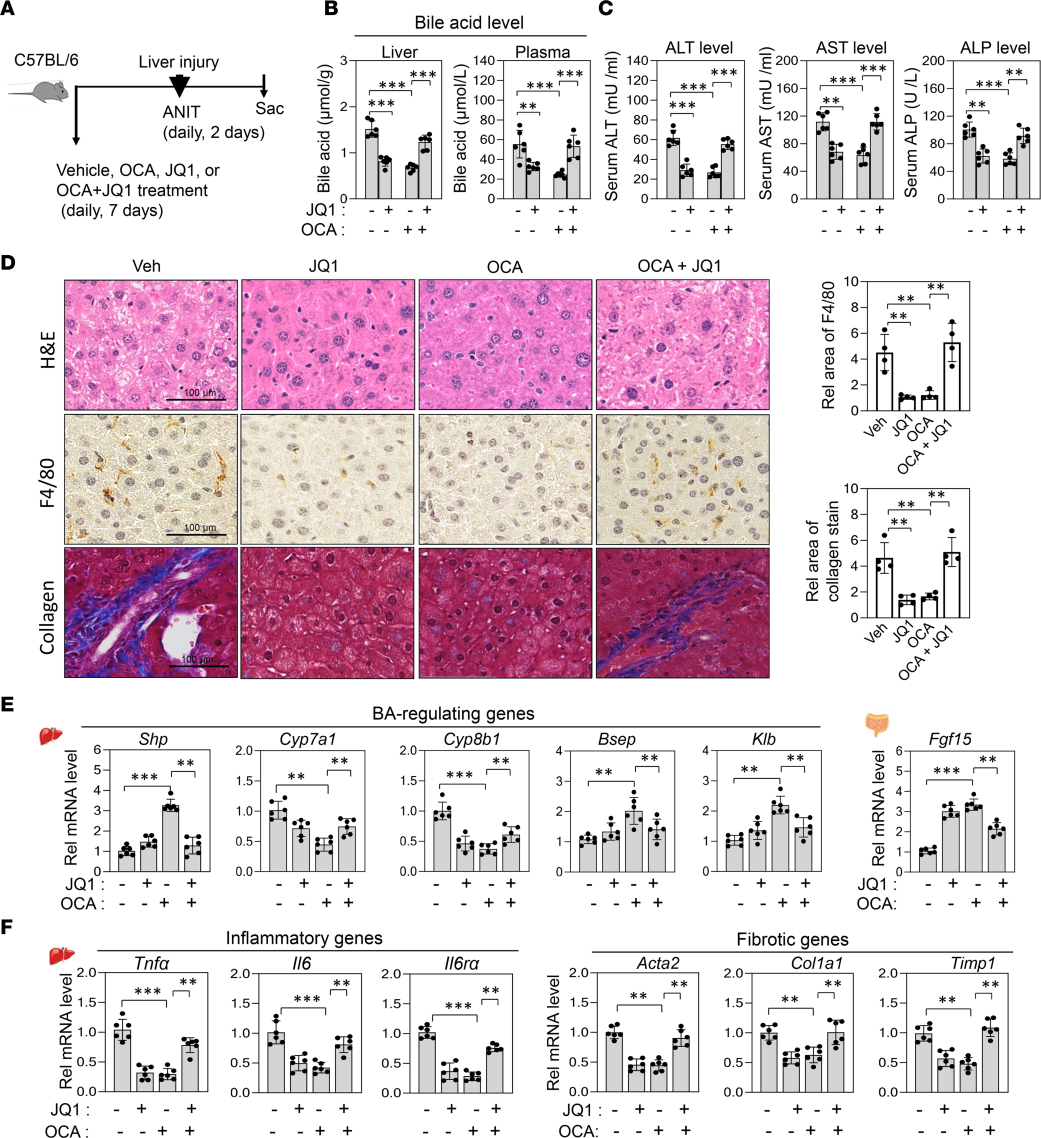
Beneficial protective effects of OCA or JQ1 in cholestatic mice are lost with cotreatment. Mice were treated daily with 10 mg/kg OCA or 50 mg/kg JQ1 for 7 days and for the last 2 days were treated daily with 35 mg/kg with ANIT. (**A**) Experimental outline. (**B** and **C**) Hepatic and plasma BA levels and serum alanine aminotransferase (ALT), aspartate aminotransferase (AST), and alkaline phosphatase (ALP) levels. (**D**) Liver sections stained with H&E to evaluate hepatic toxicity (top). Liver sections were stained with F4/80 antibody to detect macrophages (brown) (middle), and collagen (blue) was detected by staining using Masson’s trichrome method (bottom). Scale bar: 100 μm. Representative images (left) and quantitation (right). (**E** and **F**) mRNA levels of the indicated genes measured by RT-qPCR. (**B**, **C**, **E**, and **F**) Mean ± SD. Statistical significance was determined by 2-way ANOVA (*r* *=* 6 mice), **P *<* 0.01, ***P *<* 0.001.

In cholestasis, elevated BA levels in liver cause hepatotoxicity, inflammation, and fibrosis (3, 4), and activation of FXR signaling by treatment with OCA or GW4064 protects against cholestatic liver pathologies in rodent models (5, 15, 18). Inhibition of BRD4 was also shown to protect against inflammation and liver fibrosis (30, 31). We, therefore, tested whether OCA-mediated beneficial antiinflammatory, antifibrotic protective effects are enhanced by inhibition of BRD4 in cholestatic mice. FXR was activated and BRD4 was inhibited by daily treatment with OCA and JQ1, respectively, for 7 days, and then in addition for the last 2 days with α-naphthylisothiocyanate (ANIT), which induces intrahepatic cholestasis by damaging biliary epithelial cells (5, 40–43) (Figure 4A).

Treatment with either OCA or JQ1 decreased liver and serum BA levels but cotreatment reversed the effects (Figure 4B). Further, treatment with either OCA or JQ1 resulted in antiinflammatory and antifibrotic effects, which included decreased liver toxicity measured by serum ALT, AST, and ALP levels (Figure 4C), and decreased hepatocyte ballooning and degeneration, inflammation, and fibrosis as determined by histological analyses (Figure 4D, higher magnification images in Supplemental Figure 6). Consistent with these effects, OCA or JQ1 treatment increased hepatic expression of BA-regulating direct FXR target genes, *Shp*, *Bsep*, and *Klb*, and intestinal *Fgf15*, and decreased BA synthetic genes, *Cyp7a1* and *Cyp8b1* (Figure 4E). Hepatic expression of inflammatory genes, *Tnfa*, *Il6*, and *Il6ra*, and fibrotic genes and markers of hepatic stellate cell activation, such as *Acta2*, *Col1a1*, and *Timp1*, was decreased (Figure 4F). Surprisingly, the antiinflammatory and antifibrotic effects after treatment with OCA or JQ1 alone were largely lost if both drugs were administered (Figure 4, B–F). These results suggest that treatment with either OCA or JQ1 protects against cholestatic liver injury, but these beneficial effects are lost by cotreatment.

### OCA-mediated protective effects are blunted by BRD4 downregulation in cholestatic mice.

**Figure 5:**
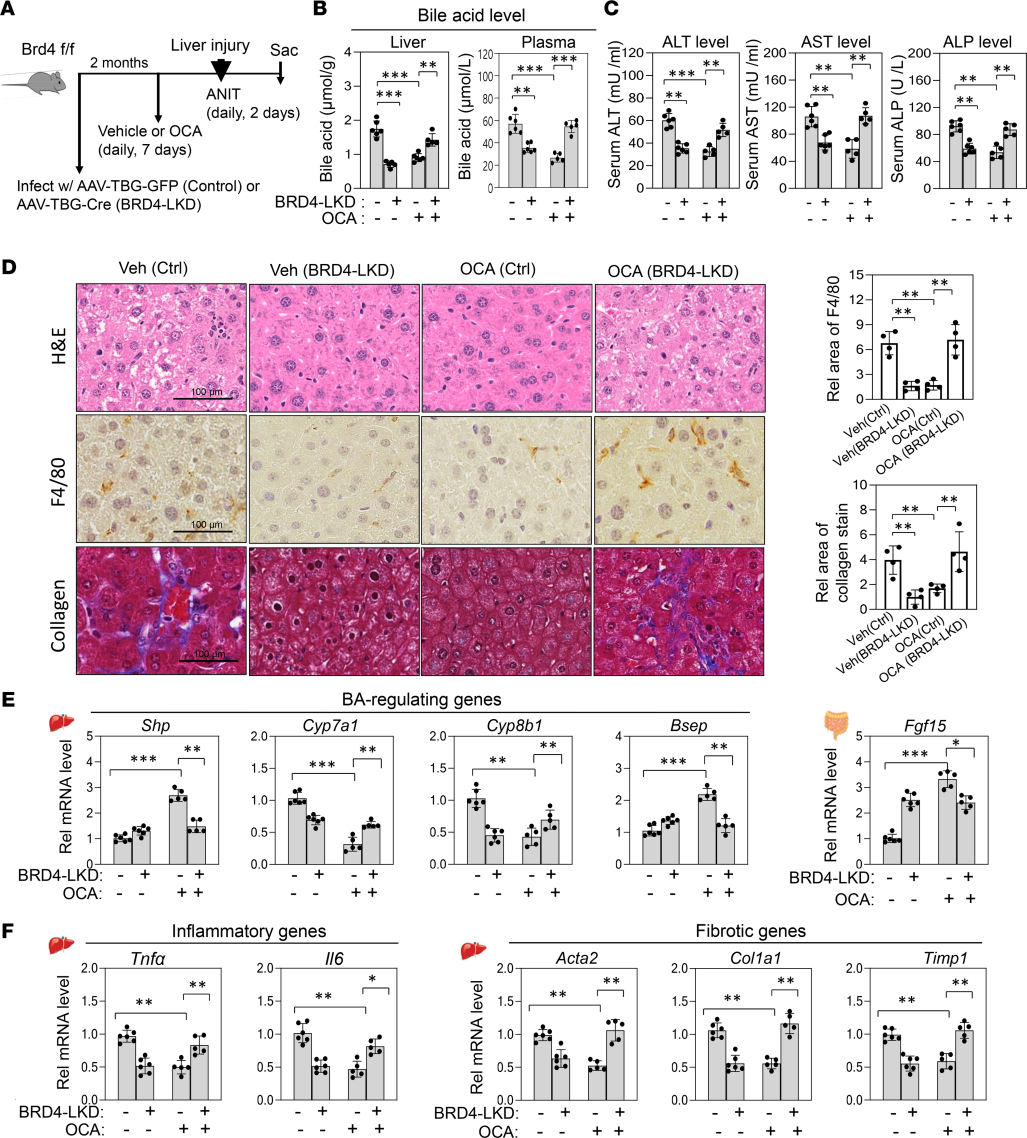
Hepatic BRD4 is required for OCA-induced beneficial protective effects against cholestasis in mice. *Brd4*-floxed mice were infected with AAV-TBG-Cre, or AAV-TBG-GFP as a control. Two months later, mice were treated daily with 10 mg/kg OCA or vehicle for 7 days and for the last 2 days were treated daily with 35 mg/kg ANIT. (**A**) Experimental outline. (**B** and **C**) Serum ALT, AST, and ALP levels and hepatic and plasma BA levels. (**D**) Representative images of liver sections stained as described in the legend to Figure 4D and quantitation by ImageJ (NIH) (right). Scale bar: 100 μm. (**E** and **F**) Levels of mRNAs of the indicated hepatic genes and of intestinal *Fgf15* measured by RT-qPCR. (**B**, **C**, **E**, and **F**) Mean and SD are plotted. Statistical significance was determined by 2-way ANOVA (*r* *=* 5–6 mice), *P *<* 0.05, **P *<* 0.01, ***P *<* 0.001.

JQ1 targets BRD4 but also inhibits other BET proteins (27). To determine the specific function of BRD4 in mediating the protective effects against cholestatic injury, hepatic BRD4 was specifically downregulated in *Brd4*-floxed mice by AAV-TGB-Cre infection for 2 months, FXR was then activated by daily treatment with OCA for 7 days, and liver injury was induced by additional treatment with ANIT for the last 2 days (Figure 5A).

Consistent with the JQ1 experiments above (Figure 4), in ANIT-treated mice, either liver-specific downregulation of BRD4 or treatment with OCA decreased levels of liver and serum BA and serum levels of ALT, AST, and ALP; ameliorated hepatotoxicity, inflammation, and fibrosis; and decreased mRNA levels of inflammatory and fibrotic genes (Figure 5, B–F, higher magnification images in Supplemental Figure 7). Remarkably, the OCA-mediated antiinflammatory and antifibrotic effects were largely lost in BRD4-downregulated mice, which is consistent with the JQ1 studies above (Figure 4). These results suggest that OCA treatment or downregulation or inhibition of BRD4 protects against cholestatic liver injury, but paradoxically, these beneficial effects are lost with combined treatment.

### Recruitment of OCA-activated FXR and silencing mediator of retinoid and thyroid hormone receptor at proinflammatory genes is BRD4 dependent.

In response to inflammatory signaling, ligand-activated FXR, as a monomer, is recruited to NF-κB–bound inflammatory genes, including IL-6 receptor subunit α (*Il6ra*) and TNF ligand superfamily member 4 (*Tnfsf4*); antagonizes NF-κB function; and represses expression of these genes, in part by recruiting the corepressors, silencing mediator of retinoid and thyroid hormone receptor (SMRT) and nuclear receptor corepressor 1 (NCoR1) (14, 15).

**Figure 6:**
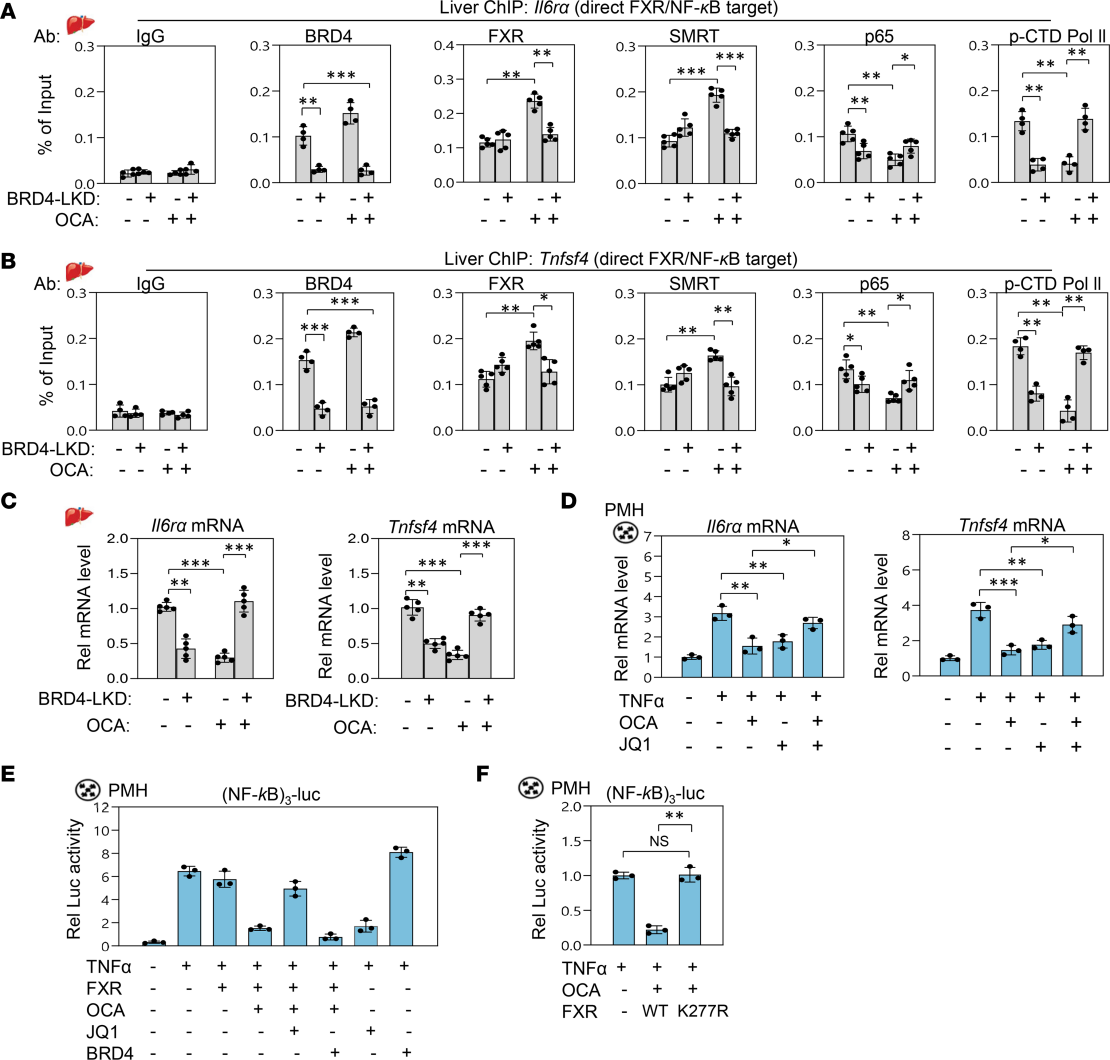
BRD4 is required for binding of OCA-activated FXR and corepressor SMRT to proinflammatory genes. (**A**–**C**) BRD4-LKD mice or control mice were treated daily with 10 mg/kg OCA or vehicle for 7 days and for the last 2 days also with 35 mg/kg ANIT. (**A** and **B**) Occupancy of the indicated factors at direct NF-κB/FXR target inflammatory genes, *Il6ra* and *Tnfsf4*, determined by liver ChIP assay and (**C**) mRNA levels of these genes measured by RT-qPCR. (**D**) PMHs were treated with 1 μM OCA, 500 nM JQ1, and 50 ng/mL TNF-α for 4 hours, and levels of *Il6ra* *and* *Tnfsf4* mRNAs were determined by RT-qPCR. (**E** and **F**) PMHs were transfected with a luciferase plasmid containing NF-κB sites and an expression plasmid for BRD4 and infected with Ad-FXR-WT or Ad-K277R-FXR. After 48 hours, cells were treated with 1 μM OCA or 500 nM JQ1 and TNF-α (50 ng/mL). Luciferase activities normalized to β-galactosidase levels. (**A**–**F**) Mean ± SD. Significance was determined by 1-way (**A**–**C**, *r* = 3–5 mice) or 2-way ANOVA (**D**–**F**, *r* *=* 3 culture dishes). *P *<* 0.05, **P *<* 0.01, ***P *<* 0.001.

To understand the molecular mechanisms by which OCA-mediated antiinflammatory effects are lost by BRD4 downregulation (Figure 5), we performed liver ChIP assays in ANIT-induced cholestatic mice to examine factor occupancy at the inflammatory genes, *Il6ra* and *Tnfsf4*, that are direct targets of both FXR and NF-κB (15). Liver-specific downregulation of BRD4 led to decreased binding of the p65 subunit of NF-κB and p-CTD Pol II at these genes (Figure 6, A and B). Treatment with OCA also decreased binding of the p65 subunit of NF-κB and p-CTD RNA Pol II but increased binding of FXR and the corepressor SMRT at the genes. However, OCA-mediated effects on binding of FXR, SMRT, NF-κB, and p-CTD Pol II were blunted by BRD4 downregulation, resulting in upregulation of *Il6ra* and *Tnfsf4* (Figure 6C).

These studies indicate that downregulation of BRD4 decreased binding of NF-κB and p-CTD Pol II at inflammatory genes, suggesting that BRD4 coactivates NF-κB inflammatory function in cholestatic liver. Surprisingly, however, downregulation of BRD4 also decreased binding of OCA-activated FXR and the corepressor SMRT, resulting in increased expression of inflammatory genes. These findings suggest that BRD4 has dual inflammatory functions in cholestatic mice. Although BRD4 has a proinflammatory function, paradoxically, antiinflammatory actions by OCA-activated FXR are also BRD4 dependent.

### BRD4 exhibits both pro- and antiinflammatory functions in PMHs.

Elevated BA levels in hepatocytes at pathological levels in cholestatic conditions were shown to initiate liver injury by triggering a hepatocyte-specific, cytokine-induced inflammatory response (44). We, therefore, tested whether the dual inflammatory actions of BRD4 observed in cholestatic mice are also observed in PMHs treated with a proinflammatory cytokine, TNF-α. Treatment with TNF-α increased expression of *Il6ra* and *Tnfsf4*, and further treatment with either JQ1 or OCA decreased expression of these genes (Figure 6D). Consistent with the results in cholestatic liver in mice (Figure 5F), OCA-mediated inhibition of inflammatory gene expression in PMHs was blunted by cotreatment with JQ1 (Figure 6D).

The role of FXR and BRD4 in NF-κB inflammatory signaling was further studied in PMHs using NF-κB promoter-luciferase reporter plasmids. Treatment with TNF-α increased NF-κB luciferase activity as expected, and treatment with JQ1 or overexpression of BRD4 decreased or increased the NF-κB activity, respectively (Figure 6E). Although overexpression of FXR without OCA did not change the luciferase activity, treatment with OCA in hepatocytes expressing FXR decreased the NF-κB activity, and these OCA-mediated effects were attenuated by treatment with the BRD4 inhibitor JQ1. These data suggest that the OCA-activated FXR inhibits NF-κB function in a BRD4-dependent manner.

In response to inflammatory signaling, agonist-activated FXR is small ubiquitin like modifier 2 (SUMO2) modified at K277, and this SUMO2 modification is critical for transrepression of NF-κB target inflammatory genes (14, 15). Consistent with these previous findings, overexpression of a SUMO2-defective K277R-FXR mutant did not decrease the NF-κB luciferase activity even after OCA treatment, suggesting that SUMOylated FXR is important for repression of inflammatory genes (Figure 6F). Together, these results from hepatocyte studies are consistent with the findings in cholestatic mice in vivo (Figure 4 and Figure 5) and establish that BRD4 does have dual inflammatory functions. Although BRD4 coactivates NF-κB function, BRD4 is also required for the repression of NF-κB activity by the OCA-activated FXR.

### OCA-mediated beneficial therapeutic effects in cholestatic mice are BRD4 dependent.

**Figure 7:**
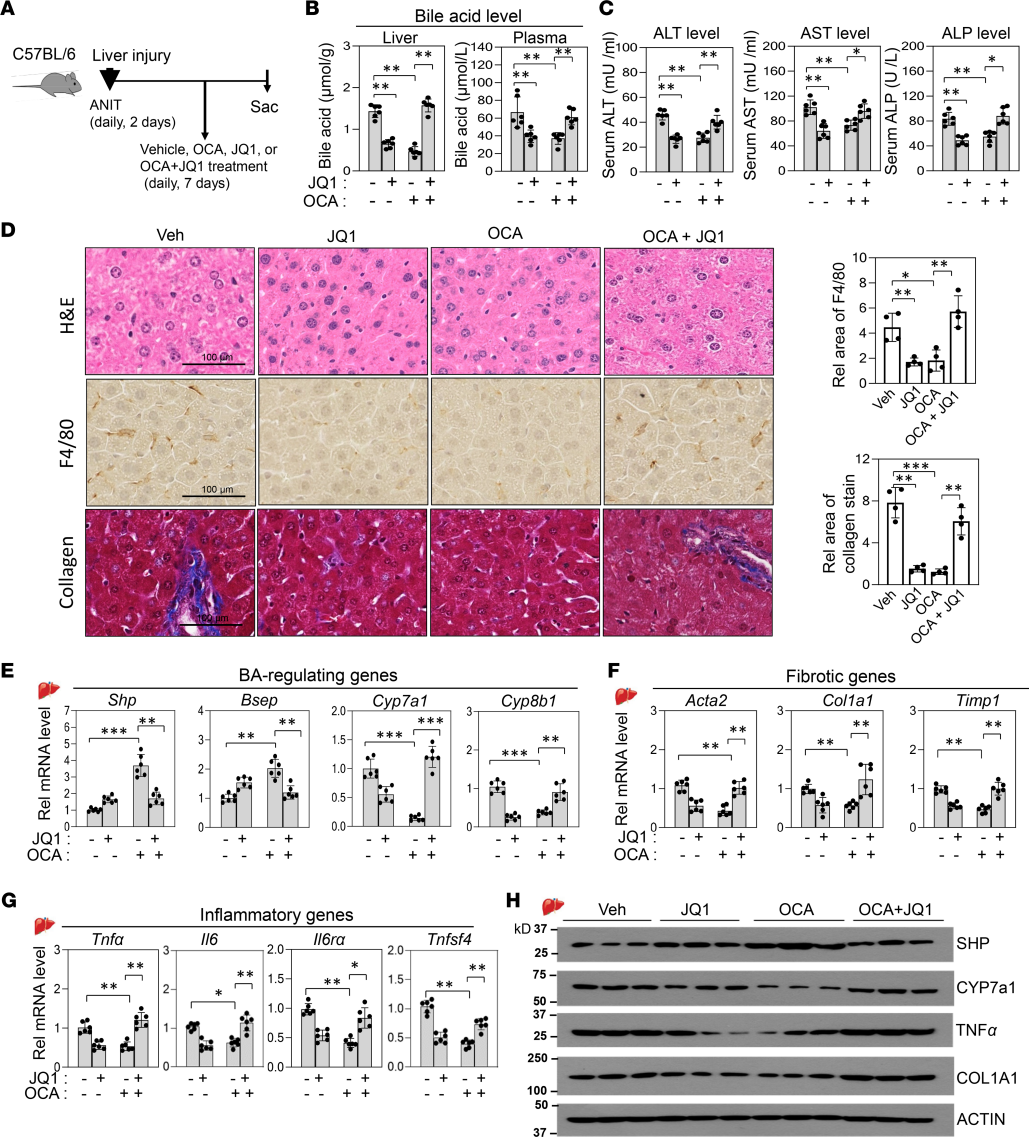
Therapeutic benefits of either OCA or JQ1 in cholestatic mice are lost with combined treatment. C57BL/6 mice were treated daily with 35 mg/kg ANIT for 2 days and further treated daily with 10 mg/kg OCA or 50 mg/kg JQ1 for 7 days. (**A**) Experimental outline. (**B** and **C**) Hepatic and plasma BA levels and serum ALT, AST, and ALP levels. (**D**) Representative images of liver sections stained as described in the legend to Figure 4D and quantitation (right). Scale bar: 100 μm. (**E**–**G**) Levels of hepatic mRNAs of the indicated genes measured by RT-qPCR. (**H**) Protein levels of SHP, CYP7A1, TNF-α, and COL1A1 in liver extracts determined by immunoblot. (**B**, **C**, and **E**–**G**) Mean ± SD are plotted. Statistical significance was determined by 2-way ANOVA (*r* *=* 6 mice), * *P* *<* 0.05, ** *P* *<* 0.01, *** *P* *<* 0.001.

We evaluated therapeutic potential of OCA and JQ1 in amelioration of cholestatic liver pathologies. Cholestatic liver injury was established first by ANIT treatment of mice for 2 days, followed by treatment for 7 days with OCA or JQ1 alone or both drugs (Figure 7A).

In cholestatic mice, treatment with either JQ1 or OCA decreased liver and serum BA levels and decreased serum levels of ALT, AST, and ALP, but these effects were severely blunted after cotreatment with both drugs (Figure 7, B and C). In histological analyses of liver sections, treatment with either OCA or JQ1 led to decreased hepatic inflammation, fibrosis, and hepatocyte ballooning (Figure 7D, higher magnification images in Supplemental Figure 8), but again, these beneficial effects of OCA or JQ1 were lost after cotreatment with both drugs. Consistent with these results, in qRT-PCR analysis, treatment with OCA increased hepatic mRNA levels of *Shp*, *Bsep*, and *Klb*, and intestinal *Fgf15*, and decreased those of BA synthetic genes, *Cyp7a1* and *Cyp8b1*, and of inflammatory and fibrotic genes, but all these OCA-mediated beneficial effects were largely abolished by cotreatment with JQ1 (Figure 7, E–G, Supplemental Figure 9). Changes in the protein levels of SHP, CYP7A1, TNF-α, and COL1A1 were consistent with the changes in mRNA levels (Figure 7H). Together, these results suggest that OCA or JQ1 treatment has therapeutic potential for cholestatic liver disease, but surprisingly, the beneficial effects are lost by cotreatment.

We next examined the effects of OCA and JQ1 in a second cholestatic mouse model. Lithocholic acid (LCA) is a toxic secondary BA that induces liver cholestasis (3, 4, 45). C57BL/6 mice were fed chow supplemented with 1% LCA for 2 days, then treated daily with either OCA or JQ1 or both drugs for 7 days (Supplemental Figure 10A). In LCA-fed cholestatic mice, treatment with either OCA or JQ1 showed beneficial effects on liver toxicity and gene expression, but treatment with JQ1 blocked the beneficial impact of OCA treatment (Supplemental Figure 10, B–F). These LCA feeding studies are consistent with the results from the ANIT-induced cholestatic mice (Figure 7) and further support the conclusion that cotreatment with OCA and JQ1 is antagonistic.

### Hepatic FXR and BRD4 mRNA levels are reduced in patients with PBC.

**Figure 8:**
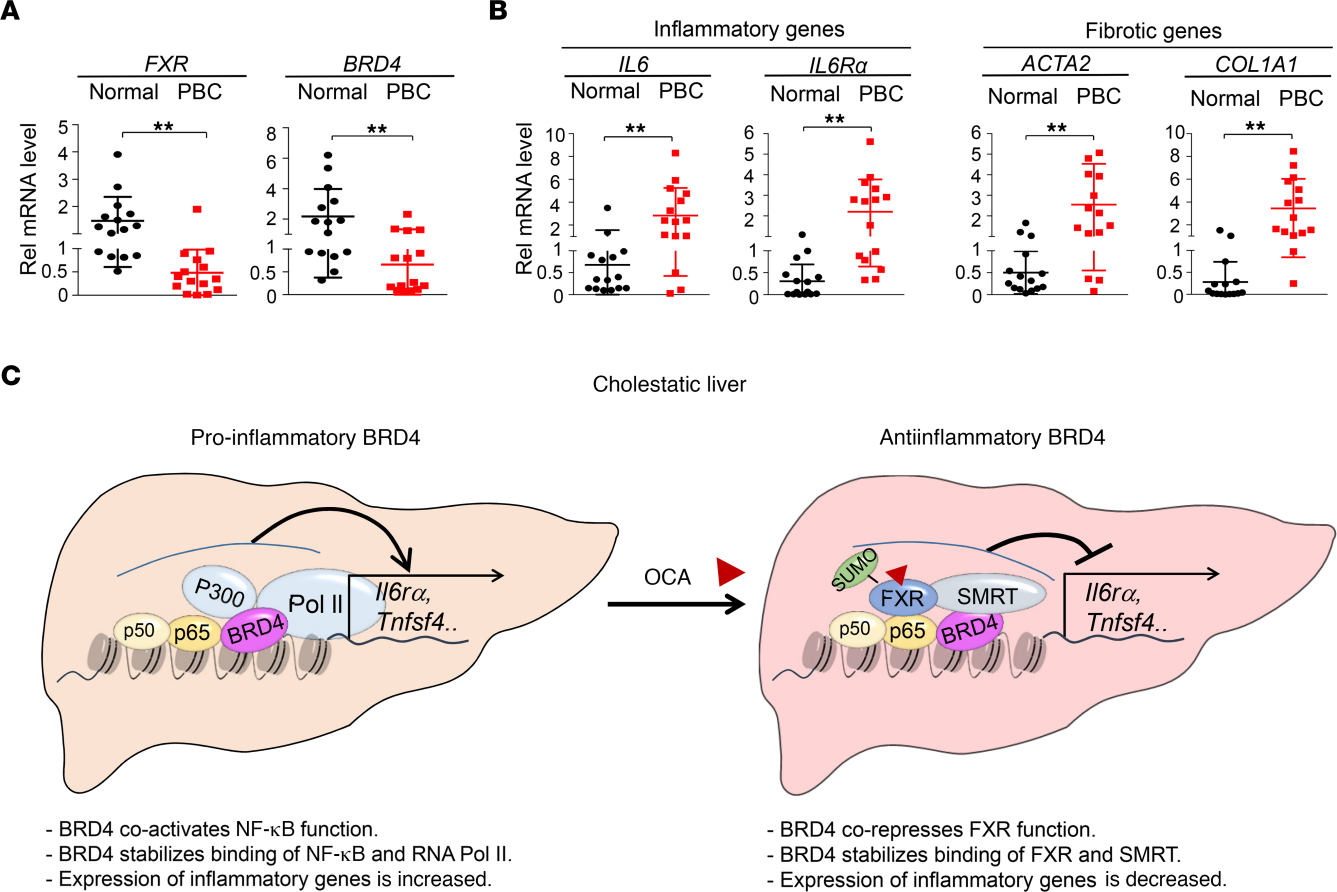
In patients with PBC, hepatic expression of *FXR* and *BRD4* is reduced and that of inflammatory and fibrotic genes is increased. (**A** and **B**) Hepatic mRNA levels of the indicated genes in 15 healthy individuals or patients with PBC were determined by RT-qPCR. Mean ± SD. Statistical significance was measured using the 1-way ANOVA. **P *<* 0.01. For statistical analysis, *r* *=* 15 individuals. (**C**) Model illustrating dual inflammatory actions of BRD4 in cholestatic liver. (Left) Proinflammatory BRD4: in cholestatic liver, BRD4 coactivates NF-κB, increasing binding of NF-κB and p-CTD Pol II at inflammatory genes and subsequently, increasing expression of the genes. (Right) Antiinflammatory BRD4: after OCA treatment, agonist-activated FXR, upon SUMOylation, binds to inflammatory genes as a monomer and recruits the corepressor SMRT, repressing expression of the genes. BRD4 is, paradoxically, required for OCA-induced antiinflammatory effects by stabilizing the binding of FXR and SMRT.

To assess potential human relevance of our findings, we examined mRNA levels of *FXR* and *BRD4* in liver samples of 15 unidentifiable patients with PBC. Hepatic mRNA levels of *FXR*, as shown previously (5), and *BRD4* were significantly reduced in PBC patients compared with healthy individuals, whereas those of inflammatory genes, *IL6* and *IL6RA*, and fibrotic genes, *ACTA2* and *COL1A1*, were all substantially elevated in the patients (Figure 8, A and B). In previous studies, hepatic mRNA and protein levels of SHP, a target of FXR/BRD4, were not changed in patients with PBC compared with healthy subjects, but nuclear levels of SHP were substantially reduced in the patients (42). These results suggest that the FXR-BRD4 function is likely dysregulated in patients with PBC.

## Discussion

In this study, we demonstrate that an epigenetic reader protein, BRD4, is a potentially novel coactivator of FXR in transcription induction of *Shp*, which is important for maintaining the normal range of BA levels in mice. We further show that BRD4 acts as a critical corepressor of agonist-activated FXR in antiinflammatory and antifibrotic actions under cholestatic conditions. Although treatment with either OCA or JQ1, a BRD4 inhibitor, has beneficial antiinflammatory antifibrotic effects in cholestatic mice, surprisingly, these beneficial effects are lost with combined treatment.

Previously, we have shown that P300 is a critical coactivator of FXR in epigenetic induction of *Shp* by catalyzing the acetylation of histone H3 at K9/14 at the *Shp* promoter (8, 10, 11). Consistent with these findings, downregulation of P300 decreased acetylated H3K9/14 levels, leading to decreased recruitment of BRD4 to the *Shp* promoter (Figure 3). Conversely, downregulation of BRD4 led to decreased binding of P300 and p-CTD Pol II, whereas FXR binding was not significantly decreased. These data support a temporal model (Figure 3G) in which agonist-activated FXR, together with RXRα, initially binds to the *Shp* promoter and recruits P300, resulting in acetylation of histone H3 at K9/14, forming a binding site for BRD4. BRD4, then, binds to the promoter and coactivates FXR, promoting stable binding of P300 and p-CTD Pol II and acetylation of histone H3 for sustained transcriptional activation of *Shp*. The results from ChIP analyses, together with biochemical and functional studies in hepatocytes and in mice, identify BRD4 as a critical coactivator of FXR in the epigenetic induction of *Shp* to maintain homeostasis in response to elevated BA levels.

As a master regulator of BA metabolism, FXR protects against BA-induced hepatotoxicity (5–7). Indeed, homozygous loss of human FXR function causes severe progressive familial intrahepatic cholestasis (46), and activation of FXR by OCA treatment protects against hepatic inflammation and fibrosis in rodent models of cholestasis, cirrhosis, and NASH (3, 4, 18). Importantly, OCA was recently approved for second-line therapy of PBC patients who did not adequately respond to UDCA (3, 4, 19). In addition to FXR, BRD4 has also emerged as an exciting potential therapeutic target for chronic liver disease and inflammatory bowel disease (29, 31), as well as cancer and neurological disorders (27, 28). In global ChIP-Seq studies, BRD4 binding sites were highly enriched at enhancer regions of numerous genes involved in fibrotic, inflammatory pathways involved in liver and cardiac fibrosis (30, 32). Consistent with these results, treatment with BET bromodomain inhibitors, such as JQ1 or I-BET151, improved liver NASH fibrosis and cardiac fibrosis in mice (30–32). In the present study, we confirmed previous findings that OCA or the BRD4 inhibitor JQ1 decreases inflammation and fibrosis in cholestatic mice, but very surprisingly, the OCA-mediated antiinflammatory antifibrotic effects were lost by cotreatment with JQ1 or downregulation of BRD4.

Molecular mechanisms for this paradoxical antagonism between JQ1 and OCA are unclear and need further investigations, but our data suggest that context-specific biphasic functions of BRD4 and FXR likely play a role. As a scaffold protein, BRD4 can interact with numerous transcription factors and coregulators to confer transcription plasticity, either activation or repression of its target genes (33). Indeed, our data suggest that BRD4 has both pro- and antiinflammatory functions in a context-specific manner (model in Figure 8C). At inflammatory genes, BRD4 coactivates NF-κB and increases expression of these genes, as previously reported (25, 30, 31). However, if OCA-activated FXR is present, BRD4 acts as a corepressor of FXR and represses transcription of the genes by stabilizing the binding of FXR and the corepressor SMRT. Similar to BRD4, ligand-activated FXR can also directly activate or repress its target genes (12, 16). In contrast to gene activation at its classical targets, including *Shp*, by an FXR/RXRα heterodimer, repression of inflammatory genes by agonist-activated FXR is mediated by a monomeric form of FXR, leading to recruitment of the corepressors, SMRT and NCoR1 (14, 15). It is likely, therefore, that gene context plays an important role and other coregulatory proteins in addition to P300 and SMRT are involved.

Previous studies have shown that a monomeric SUMOylated form of FXR antagonizes NF-κB functions in mouse models of NASH and colitis (14, 15). Based on the results from our hepatocyte studies, SUMOylated FXR likely acts in a similar manner in cholestatic liver in mice since overexpression of OCA-activated FXR-WT, but not SUMO-defective K277R-FXR, inhibited NF-κB luciferase activity in a BRD4-dependent manner. Further studies will be needed to determine whether our findings on the hepatoprotective role of the FXR/BRD4 complex in cholestatic mice can be extended to the NASH and colitis models. In addition to FXR, SUMO modification of agonist-activated nuclear receptors, such as PPARγ, LXRα/β, and PXR, was also shown to repress acute and/or chronic inflammation (47–49). It will be, thus, interesting to see whether cotreatment with a BET protein inhibitor and an agonist for these receptors has the same antagonistic effects in treatment of inflammatory disease as we have seen in the present OCA-activated FXR study.

Hepatic expression of FXR was shown to be reduced in PBC patients compared with healthy subjects (5). We confirmed this previous finding but also observed that mRNA levels of BRD4 were significantly reduced in the patients. Consistent with our data that OCA activation of FXR or JQ1 inhibition of BRD4 downregulated inflammatory and fibrotic genes in cholestatic mice, mRNA levels of inflammatory genes *IL6* and *IL6RA* and fibrotic genes *ACTA2* and *COL1A1* were all highly elevated in the patients. Although from a correlative, small-scale gene expression study, and requiring confirmation in larger studies, our findings suggest that FXR/BRD4 function is likely dysregulated in the PBC patients and that this impaired function may contribute to inflammatory and fibrotic pathologies in the patients.

In conclusion, we identify BRD4 as a potentially novel transcription partner of FXR in maintaining BA homeostasis and protecting against cholestatic liver injury. The BET family proteins, including BRD4, have received great interest because of their therapeutic potential for treating inflammatory fibrotic diseases, including cancer and neurological and metabolic disorders (24, 27–32). Given the beneficial effects of JQ1 and OCA, acting by different mechanisms, in treating liver diseases, combined therapy with these drugs might be expected to enhance therapeutic effect. Paradoxically, however, we show that cotreatment with JQ1 and OCA results in antagonism in cholestatic mice. These unexpected findings suggest that combination therapy of liver disease patients with inflammation and fibrosis complications with both the FXR agonist OCA and a BET protein inhibitor like JQ1 may lead to unfavorable clinical outcomes.

## Methods

### Materials and reagents.

Information on antibodies is provided in Supplemental Table 1. ON-TARGET*plus* mouse siRNAs for *Brd4* (L-041493) and *Ep300* (L-003486) were purchased from Dharmacon; AAV8-TBG-Cre (VB1724) and AAV8-TBG-GFP (VB1743) from Vector Biosystems; GW4064 (HY-50108), OCA (HY-12222), and JQ1 (HY-13030) from MedChemExpress; ANIT (N4525) from MilliporeSigma; and TNF-α (ab9642) from Abcam.

### Animal studies.

*Brd4*-floxed mice have been described previously and were generated and maintained in-house (22). For LKD of BRD4, 8-week-old *Brd4*-floxed male mice were injected with 3 × 10^11^ genome copies/body weight of AAV8-TBG-Cre as a control or AAV8-TBG-GFP via the tail vein as described previously (5, 34, 50). To examine the protective effects of FXR activation or BRD4 inhibition on drug-induced intrahepatic cholestasis, C57BL/6 mice or BRD4-LKD mice were treated daily for 7 days by oral gavage with 10 mg/kg OCA or i.p. with 50 mg/kg JQ1 or both drugs and for the last 2 days were treated daily by oral gavage with 35 mg/kg ANIT. Alternatively, BRD4-LKD mice were treated for 7 days with OCA and with ANIT for the last 2 days. To examine the therapeutic potential of OCA and JQ1, mice were treated daily with ANIT or fed 1% LCA-supplemented chow (Dyets Inc.) for 2 days and then further treated daily with either 10 mg/kg OCA or 50 mg/kg JQ1 for 7 days under normal chow. Plasma ALP (ab 83369, Abcam), ALT (MAK052, MilliporeSigma), and AST (MAK055, MilliporeSigma) levels and BA levels (DZ042A, Diazyme) from liver and plasma were measured using commercial kits. To measure gallbladder volume, the intact gallbladder was transferred to a tube containing 100 μL PBS and punctured with a 22G needle, and the sample was centrifuged at 6000*g* for 5 minutes at room temperature. The volume of the supernatant minus the 100 μL is reported as the gallbladder volume.

### PMHs.

PMHs were isolated by collagenase (0.8 mg/mL, MilliporeSigma) perfusion as described (34, 50) and cultured in M199 medium (M3769, MilliporeSigma) containing 10% fetal bovine serum. To downregulate or inhibit BRD4, PMHs were transfected using Lipofectamine RNAiMAX Transfection Reagent (Thermo Fisher Scientific) with *Brd4* siRNA (siBrd4, 50 nM) for 72 hours or treated with JQ1 (500 nM) for 24 hours, respectively. To activate FXR signaling, the cells were treated with 1 μM GW4064 in corn oil or vehicle or 1 μM OCA in 1% methylcellulose or vehicle for 3–5 hours. To induce inflammation, PMHs were treated with a proinflammatory cytokine, TNF-α (50 ng/mL), or vehicle (PBS) as a control for 4 hours.

### RT-qPCR.

Total RNA was isolated using TRIzol (Invitrogen, Thermo Fisher Scientific) and levels of mRNA were measured. Primers used are listed in Supplemental Table 2.

### Co-IP.

Co-IP assays were done as described previously (37, 50). Briefly, whole-cell extracts in co-IP buffer (50 mM Tris-HCl, pH 8.0, 150 mM NaCl, 2 mM EDTA, 0.5% NP40, 5% glycerol) from livers of mice treated with either 30 mg/kg GW4046 or 10 mg/kg OCA for 1–3 hours were incubated with 2 μg of FXR antibody or control IgG for 2 hours, and the immune complex was collected by incubation with a 25% protein G-sepharose (GE Healthcare) slurry. After 2 hours, sepharose beads were washed 3 times with co-IP buffer, and bound proteins were detected by immunoblotting using antibodies against BRD2, BRD3, or BRD4.

### Luciferase reporter assays.

HepG2 cells (ATCC, HB-8065) or PMHs were transfected with 200 ng of a luciferase promoter-reporter plasmid containing the *Shp* promoter (*Shp-luc*) or containing 3 copies of a NF-κB site [*(NF-κB)_3_-luc*], respectively, and a plasmid for β-galactosidase (200 ng). For *Shp* promoter studies, cells were infected with Ad-FXR and transfected with 50 nM siBrd4 and 72 hours later were treated with 1 μM GW4064 for 3 hours. Alternatively, cells were infected with Ad-FXR, and after 48 hours, were treated with 500 nM JQ1 followed 24 hours later by treatment with 1 μM GW4064 for 3 hours. For FXR-SUMO studies, PMHs were transfected with an expression vector for BRD4 or infected with Ad-FXR-WT or Ad-FXR-K277R expressing a SUMO2-defective mutant (15), and 48 hours later, cells were treated with 500 nM JQ1 for 24 hours, 1 μM OCA for 4 hours, or 50 ng/mL TNF-α for 4 hours; luciferase activities were normalized to β-galactosidase activities.

### ChIP.

ChIP assays were done as described previously (37, 50). Briefly, minced mouse liver or PMHs were washed twice with PBS, then incubated with 1% formaldehyde for 10 minutes at 37°C. Glycine was added to a final concentration of 125 mM, and the sample was incubated with shaking for 5 min at room temperature. The samples were resuspended in hypotonic buffer and cells were lysed by homogenization. Nuclei were isolated by centrifugation and resuspended in sonication buffer (50 mM Tris-HCl, pH 8.0, 2 mM EDTA, and 1% SDS). The samples were sonicated 4 times at 10-second intervals using a Qsonica XL-2000 instrument at power output setting 8. The chromatin sample (300 μL) was precleared by incubation with protein G-sepharose slurry for 1 hour and immunoprecipitated using 1 μg to 1.5 μg of antibody overnight at 4°C. The immune complexes were collected by incubation with a protein G-sepharose slurry for 1 hour, and the beads were washed with 0.1% SDS, 1% Triton X-100, 2 mM EDTA, and 20 mM Tris-HCl, pH 8.0, 3 times containing successively 150 mM NaCl, 500 mM NaCl, or 0.25 M LiCl. Then, bound chromatin was eluted and incubated overnight at 65°C to reverse the cross-linking. DNA was isolated for quantitative PCR. For re-ChIP, the immune complex was eluted with 10 mM DTT at 37°C for 30 minutes, diluted 20× with 20 mM Tris-HCl at pH 8.0, 150 mM NaCl, 2 mM EDTA, and 1% Triton X-100 and then reimmunoprecipitated. Primer sequences for ChIP–quantitative PCR are in Supplemental Table 2.

### IHC.

Paraffin-embedded liver sections were incubated with F4/80 antibody, and the antibody was detected by IHC staining using peroxidase-based detection (ab64238, Abcam). Liver collagen was detected with a Trichrome Stain Kit (ab150686, Abcam). Liver sections were imaged with a NanoZoomer Scanner, and ImageJ software was used for image analysis and quantification.

### Human PBC patients.

Liver specimens from 15 unidentifiable PBC patients or control individuals (organ donors) were obtained from the Liver Tissue Cell Distribution System, and mRNA levels of selected genes were quantified by RT-qPCR.

### Statistics.

GraphPad Prism 8 (GraphPad Software Inc) was utilized for data analysis. Statistical analysis of different groups was performed using the Student’s unpaired 1-tailed *t* test or 1- or 2-way ANOVA as appropriate. Differences with *P* *<* 0.05 were considered statistically significant.

### Study approval.

All animal studies and biosafety protocols were approved by the Institutional Animal Use and Care and Biosafety Committees of the University of Illinois at Urbana-Champaign and were in accordance with NIH guidelines. Human liver samples were not collected specifically for this study, and no one on our study team has access to the subject identifiers linked to the specimens or data. Thus this study is not considered human subjects research, and ethical approval was not required (see §46.104 in Part 46—Protection of Human Subjects in the Electronic Code of Federal Regulations).

## Author contributions

HJ, JC, LFC, and JK designed research; HJ, JC, XH, and HS performed experiments; SYW and CMC provided critical reagents; HJ, JC, XH, HS, BK, LFC, and JKK analyzed data; and HJ, BK, LFC, and JKK wrote the paper.

## Supplementary Material

Supplemental data

## Version 1 12/08/2020

In-Press Preview

## Version 2 01/11/2021

Electronic publication
