# Supplementary material for: BRD4 inhibition and FXR activation, individually beneficial in cholestasis, are antagonistic in combination
Source: JCI Insight. 2020 Dec 8;6(1):e141640. doi: 10.1172/jci.insight.141640 (PMC7821603; doi:10.1172/jci.insight.141640)
Supplement: Supplemental data [file jciinsight-6-141640-s073.pdf]

Supplemental Information

BRD4 inhibition and FXR activation, individually beneficial in cholestasis, are antagonistic in combination

Jung *et al.*

Supplemental Figures

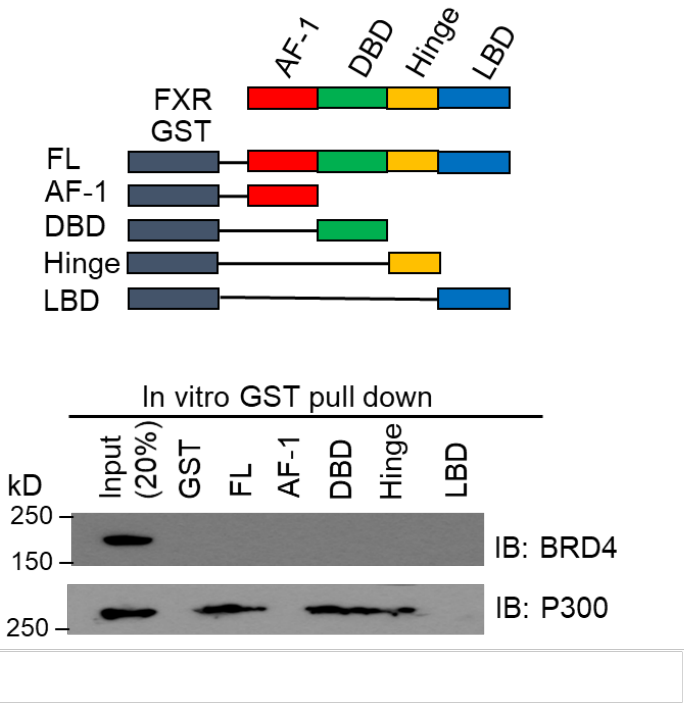

Supplemental Figure 1. BRD4 and FXR do not directly interact. GST-pull down: Schematic of the GST fusion proteins containing different regions of FXR (top). GST-FXR fusion proteins expressed in bacteria were purified by Glutathione Sepharose-4B slurry (GE Healthcare, Inc) and 1-2 μg of GST-FXR fusion proteins were incubated with p300 or BRD4 synthesized using the in vitro transcription and translation kit (Promega, Inc) and p300 or BRD4 bound to the GST-FXR proteins was detected by Immunoblotting (bottom). Consistent results were obtained from 3 independent GST pull down assays.

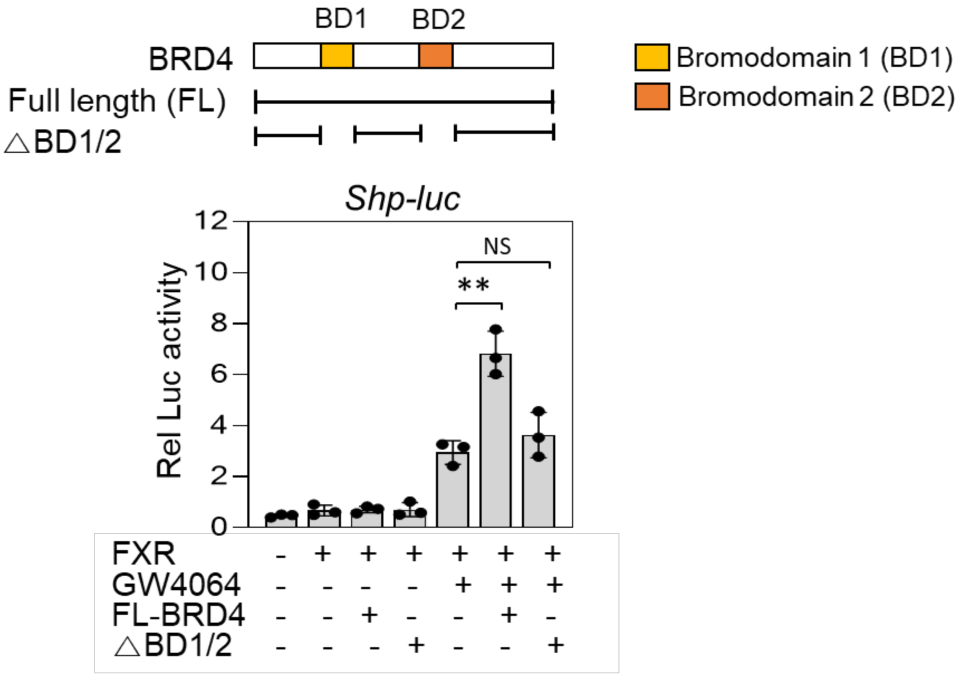

Supplemental Figure 2. The BD1 and BD2 domains are important for FXR coactivation of *Shp* by BRD4. HepG2 cells were infected with Ad-FXR or transfected with expression plasmids for full length BRD4 (FL-BRD4) or BRD4 lacking the BD1 and BD2 domains (∆BD1/2) and reporter plasmids, *Shp-luc* and β-galactosidase, and then, treated with GW4064, as indicated. Luciferase activities normalized to β-galactosidase levels. Mean and standard deviation are plotted. Statistical significance was determined by one-way ANOVA (n=3 culture dishes). * $p < 0.05$, ** $p < 0.01$, NS, not significant. Consistent results were obtained from 2 independent experiments.

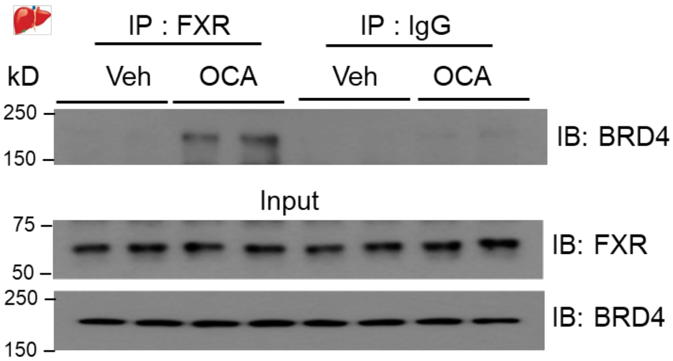

Supplemental Figure 3. OCA treatment of mice increases the interaction of BRD4 with the FXR complex in liver extracts. C57BL/6 mice were treated with OCA or vehicle for 3 h and CoIP assays were done. Levels of BRD4 in anti-FXR immune complexes from whole cell liver extracts and of input proteins determined by immunoblotting. Tissue from 2 mice were pooled for each sample and the experiment was done twice.

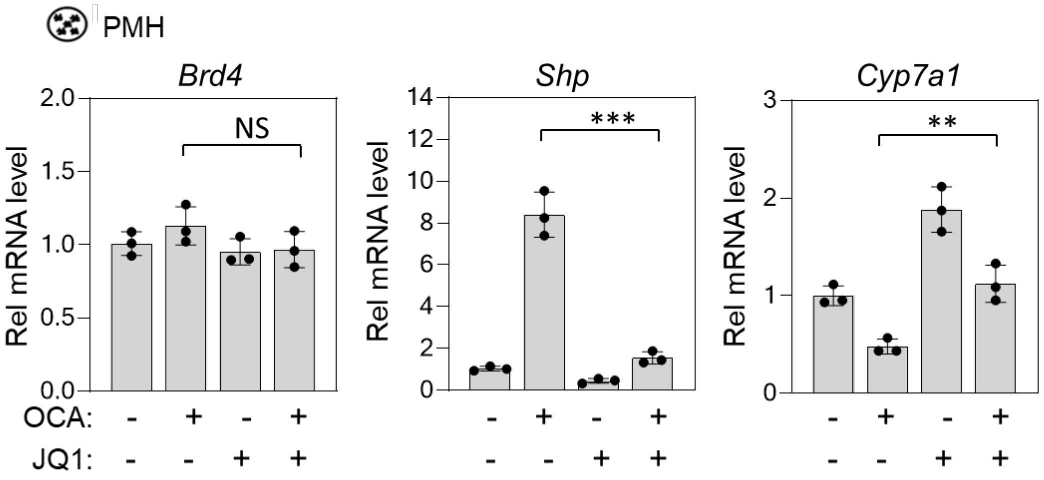

Supplemental Figure 4. OCA-mediated induction of *Shp* and repression of *Cyp7a1* are BRD4-dependent. OCA treatment increased *Shp* mRNA levels and subsequently, decreased *Cyp7a1* mRNA levels, and these effects were blunted by treatment with the BRD4 inhibitor, JQ1. PMH were treated with either 1 µM OCA or 500 nM JQ1 for 3 h or 24 h, respectively, or both. Levels of *Shp and Cyp7a1* mRNAs determined by RT-qPCR. Mean and standard deviation are plotted. Statistical significance was determined by two-way ANOVA (n=3 culture dishes). * $p < 0.05$, ** $p < 0.01$, *** $p < 0.001$, NS, not significant. Consistent results were obtained from 2 independent experiments.

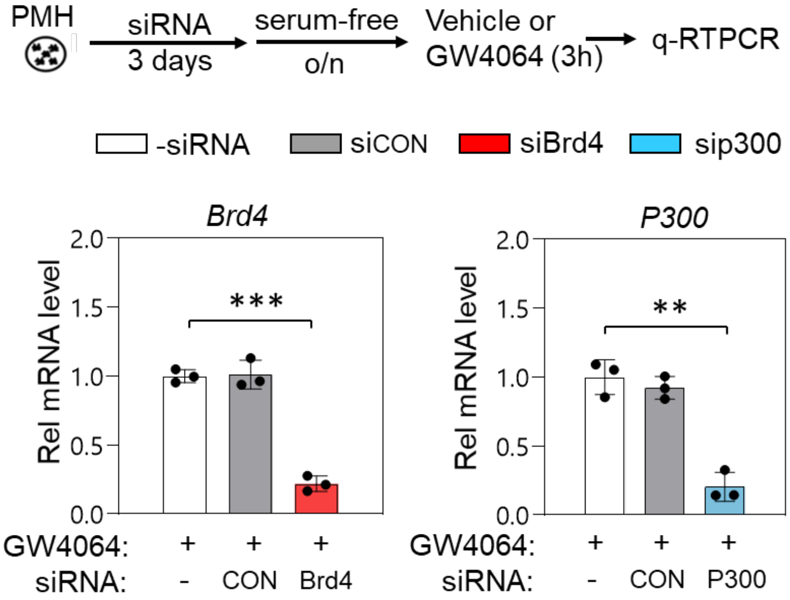

Supplemental Figure 5. siRNA-mediated downregulation of BRD4 or p300 in primary mouse hepatocytes (PMH). To examine the effects of downregulation of BRD4 or p300 on factor occupancy (Fig. 3D) and H3K9/14-Ac levels (Fig. 3E) at the *Shp* promoter in PMH, BRD4 or p300 was downregulated using siRNA. Experimental outline (top) and mRNA levels of *Brd4* (bottom, left) and *p300* (bottom, right). Mean ± SD. Statistical significance was determined by one-way ANOVA **(**n=3 culture dishes**)**, ** $p < 0.01$, *** $p < 0.001$. Consistent results were obtained from 2 independent experiments.

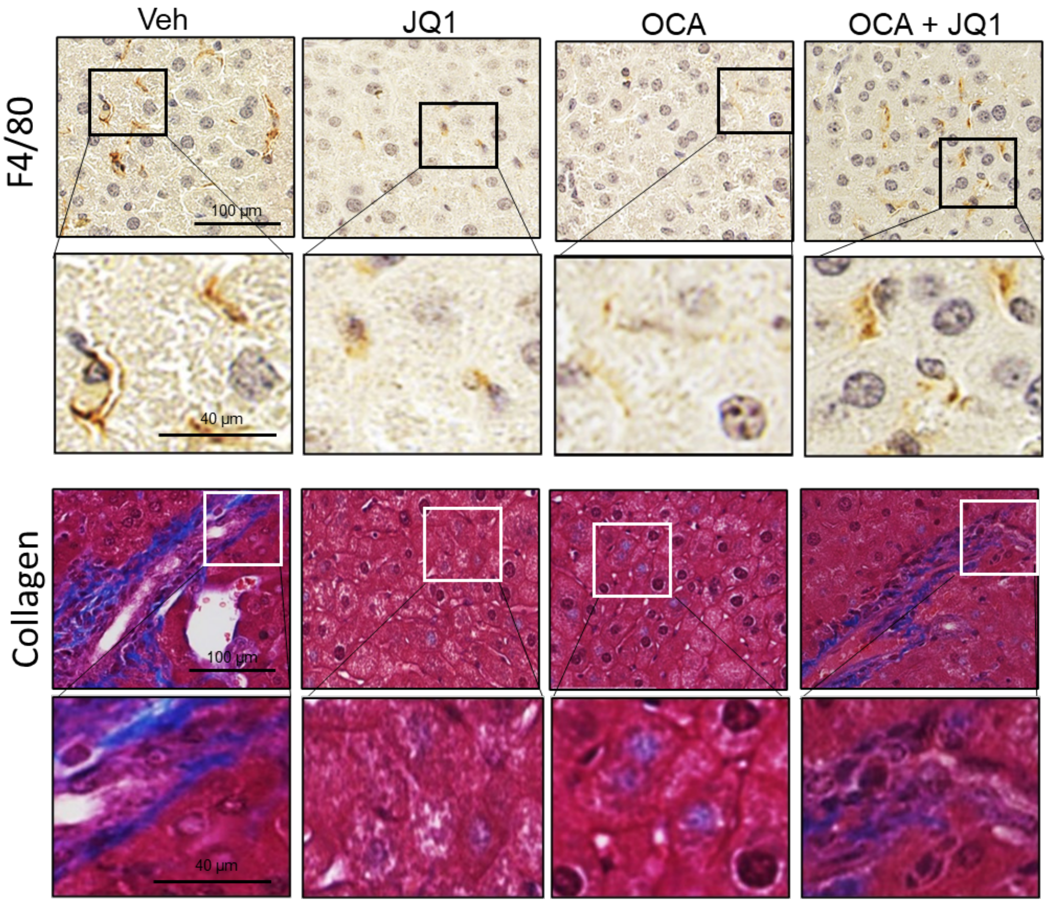

Supplemental Figure 6. Images from Figure 4D at higher magnification showing F4/80 (top) and collagen (bottom) staining of liver sections (scale bar = 100 μm in the original images and 40 μm in the images at increased magnification).

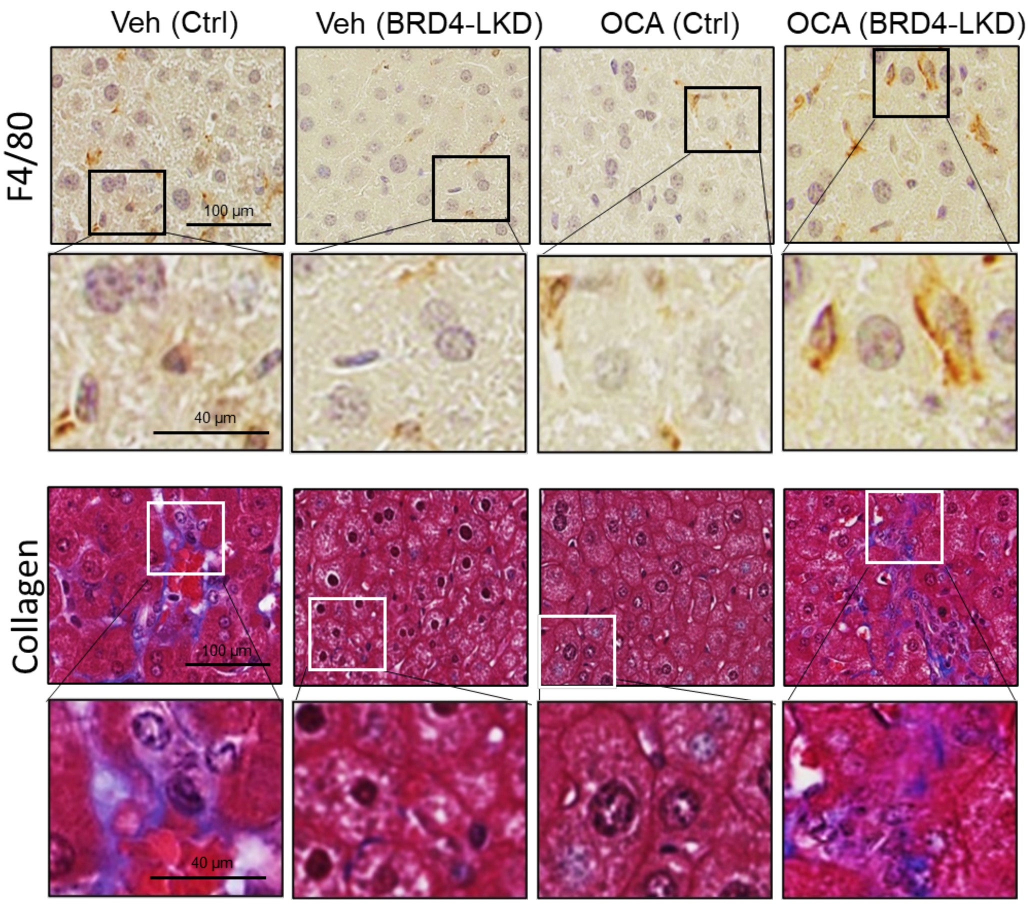

Supplemental Figure 7. Images from Figure 5D at higher magnification showing F4/80 (top) and collagen (bottom) staining of liver sections (scale bar = 100 μm in the original images and 40 μm in the images at increased magnification).

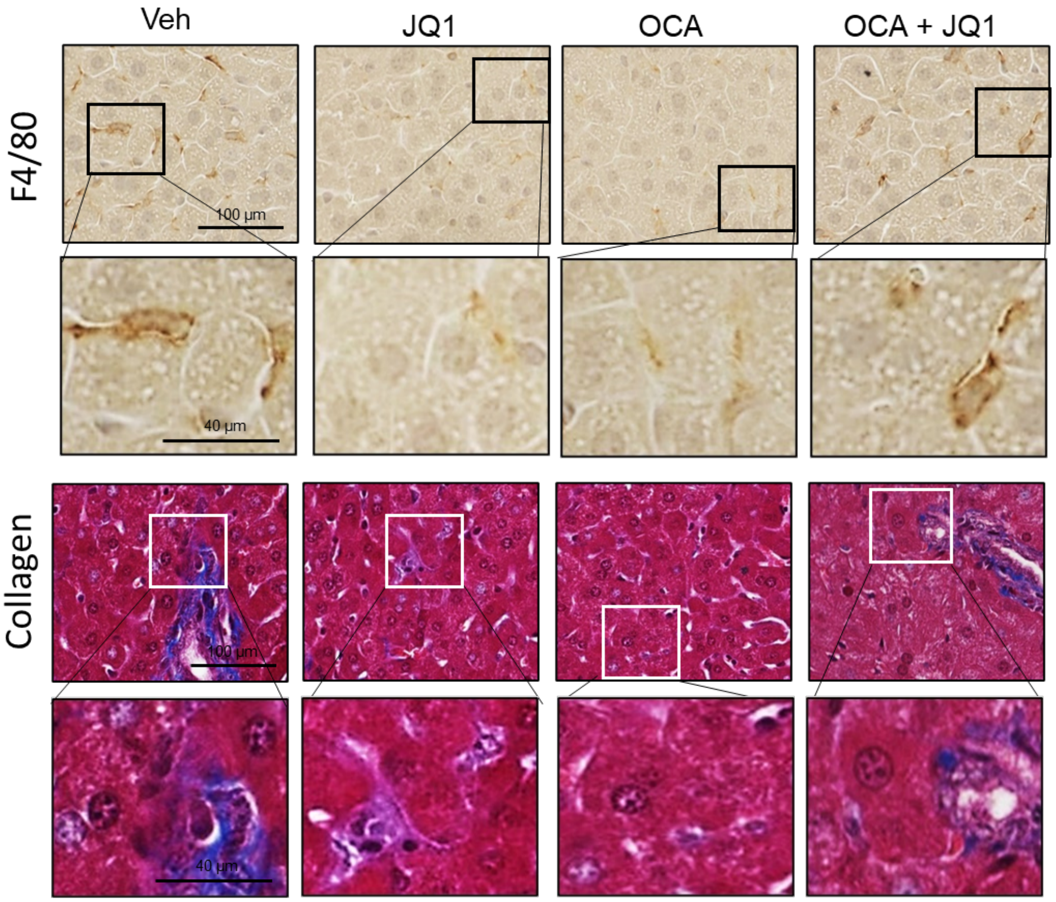

Supplemental Figure 8. Images from Figure 7D at higher magnification showing F4/80 (top) and collagen (bottom) staining of liver sections (scale bar = 100 μm in the original images and 40 μm in the images at increased magnification).

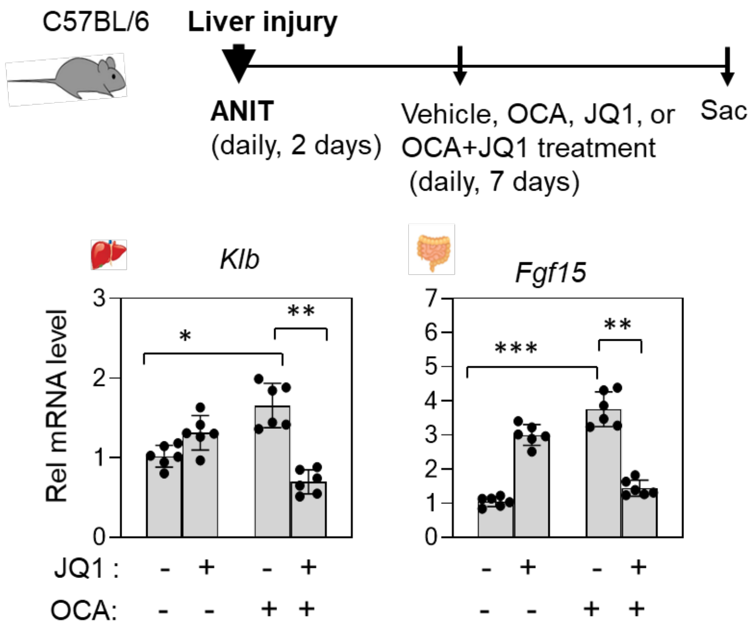

Supplemental Figure 9. Effects of treatment with either OCA or JQ1, or cotreatment with both drugs on mRNA levels of hepatic *Klb* and intestinal *Fgf15*. C57BL/6 mice were treated daily with 35 mg/kg with ANIT for 2 days and then, further treated daily with 10 mg/kg OCA or 50 mg/kg JQ1 for 7 days. Levels of hepatic and intestinal mRNAs of the indicated genes measured by RT-qPCR. Mean and standard deviation are plotted. Statistical significance was determined by two-way ANOVA (n=6 mice), ** p < 0.01, *** p < 0.001.

Cholestatic Liver Injury by Lithocholic acid (LCA) Feeding

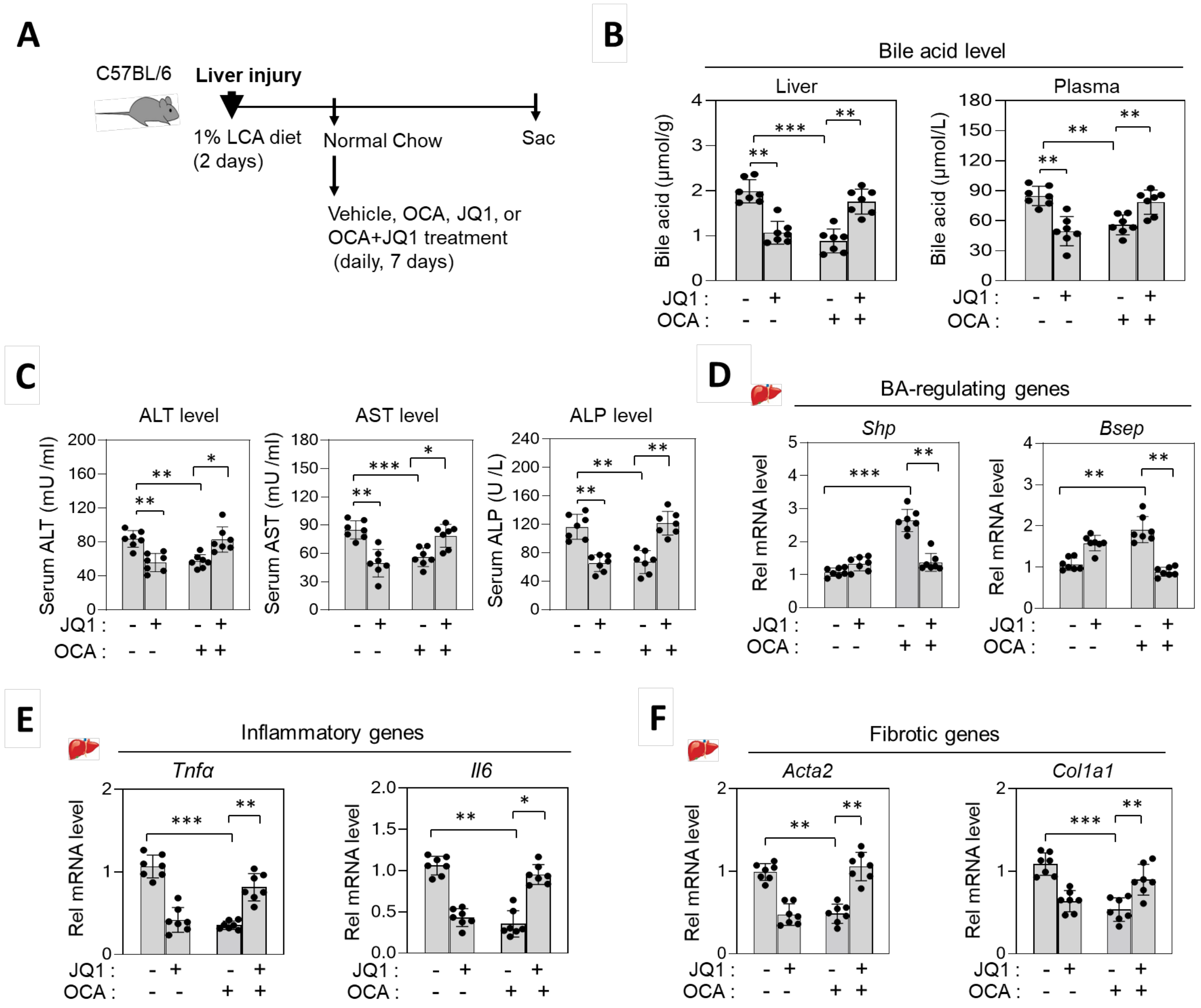

Supplemental Figure 10. In LCA-induced cholestatic mice, potential therapeutic benefits of OCA or JQ1 are largely abolished by co-treatment with both drugs. C57BL/6 mice were fed with 1% LCA-supplemented chow for 2 days and treated daily with either 10 mg/kg OCA or 50 mg/kg JQ1 or both for 7 days under normal chow conditions. **(A)** Experimental outline. **(B)** Levels of hepatic and plasma BA. **(C)** Serum levels of ALT, AST, and ALP. **(D, E, F)** Hepatic mRNAs of the indicated genes measured by RT-qPCR. **(B-F)** Mean and standard deviation are plotted. Statistical significance was determined by two-way ANOVA (n=7 mice), * $p < 0.05$, ** $p < 0.01$, *** $p < 0.001$.

Supplemental Table 1. Antibody information

**Name**	**Supplier**	**Catalog number**	**Dilution**
Histone H3	Abcam	ab1791	ChIP (1μg)
BRD4	BETHYL Lab	A301-985A	ChIP (1.5μg), IB (1:2000)
FXR	Santa Cruz	sc-25309	ChIP (1.5μg), IB (1:2000)
P300	Santa Cruz	sc-48343	ChIP (1μg)
Pol II	Abcam	ab5131	ChIP (1μg)
BRD2	BETHYL Lab	A302-583A	IB (1:4000)
BRD3	Santa Cruz	sc-81202	IB (1:4000)
NF-κB p65	Cell Signaling	8242	ChIP(1μg)
Flag M2	Sigma	F1804	IB (1:3000)
F4/80	NOVUS Biologicals	NB600-404	IHC (1:2000)
AcH3(K9/K14)	Cell Signaling	9677	ChIP (1μg)
B-ACTIN	Cell Signaling	4970	IB (1:10000)
SMRT	Abcam	ab24551	ChIP (1μg)
SHP	Abcam	Ab186874	IB (1:3000)
CYP7A1	Thermo Fisher	PA5-100892	IB (1:4000)
TNFα	Abcam	ab183218	IB (1:3000)
COL1a1	Santa Cruz	sc-293182	IB (1:4000)

ChIP volume : 300μl

Supplemental Table 2. Primers used for RT-qPCR and ChIP-qPCR

**RT-qPCR**	Forward (5'-3')	Reverse (5'-3')
Shp	CAAGAAGATTCTGCTGGAGG	GGATGTCAACATCTCCAATG
Bsep	CAATGTTCAGTTCCTCCGTTCA	TTTGGTGTTGTCCCCSTSCTTG
Mrp2	TATCCCCGGGAAATCTGTTC	TAACCAACATTCTCCGCGC
Cyp7a1	AACGGGTTGATTCCATACCTGG	GTGGACATATTTCCCCATCAGTT
Cyp8b1	GAATCTAACCAGGCCATGCT	AGGAGCTGGCACCTAGACT
Oatp	GTCTTACGAGTGTGCTCCAGAT	GGAATACTGCCTCTGAAGTGGATT
Ntcp	TACCTCCTCCCTGATGCCTTTC	TGCGTCTGCAGCTTGGATTTA
Fgf15	GTTTCACCGCTCCTTCTTTG	CATCCTCCACCATCCTGAAC
Ostα	TGGACCCTGGAAGACATA	TAACCACTGATAAGGCTGAG
Ostβ	ATCTTGATGACTCCATAATG	GTCTTTCTCTTTCAACTCA
Asbt	TGGAATGCAGAACACTCAGC	GCAAAGACGAGCTGGAAAAC
Cck	AAGTGACCGGGACTACATGG	CCCACTACGATGGGTATTCG
Mafg	GACCCCCAATAAAGGAAACAA	TCAACTCTCGCACCGACAT
Lsd1	AGCAGCCCTGTTTCCCAGAC	TGCAATGTGCGATTCCTGAT
Klb	CGAGCCCATTGTTACCTTGT	CTCCAAAGGTCTGGAAGCAG
Fxr	TGTGAGGGCTGCAAAGGTT	ACATCCCCATCTTGGAC
Tnfα	AGCCCCCAGTCTGTATCCTT	GGTCACTGTCCCAGCATCTT
Il6	AGAAGGAGTGGCTAAGGACCAA	AACGCACTAGGTTTGCCGAGTA
Il6rα	CCAGGTGCCCTGTCAGTATT	CTGGACTTGCTTCCCACACT
Tnfsf4	CTGAACGATGGTCGAAGGAT	ACAACAATCAGCTCCCCATC
Acta2	CAGCCATCTTTCATTGGGATGGAG	AATGCCTGGGTACATGGTGG
Col1a1	GAACTGGACTGTCCCAACCC	TTGGGTCCCTCGACTCCTAC
Timp1	ACTCGGACCTGGTCATAAGGG	CGCTGGTATAAGGTGGTCTCGT

**ChIP**	Forward (5'-3')	Reverse (5'-3')
Shp (-412/-100)	CAGTGAGAACCCTGGTCTT	CTGGCCAAACAACCTTGAC
Shp (-3.0/-2.7kb)	ACAGACAAGGCCACACTTCC	TGGCTCAACAGGTAAGAGCA
Il6rα	GGTACCCACAGATCCCAGAA	GTTGCTGAGGACCAAGTTGC
Tnfsf4	AAATTTTGGTGCATGTGTGTG	GCCAAGCCTGATAACCTGAG
